# Fentanyl for labour pain management: a scoping review

**DOI:** 10.1186/s12884-022-05169-x

**Published:** 2022-11-17

**Authors:** Kyaw Lwin Show, Chetta Ngamjarus, Kiattisak Kongwattanakul, Siwanon Rattanakanokchai, Chatuporn Duangkum, Meghan A. Bohren, Ana Pilar Betrán, Monsicha Somjit, Wint Ye Hla Win, Pisake Lumbiganon

**Affiliations:** 1 Doctor of Epidemiology and Biostatistics Program, Department of Epidemiology and Biostatistics, Khon Kaen University, Khon Kaen, Thailand; 2 Department of Medical Research, Ministry of Health, Yangon, Myanmar; 3 Department of Epidemiology and Biostatistics, Faculty of Public Health, Khon Kaen University, Khon Kaen, Thailand; 4 Department of Obstetrics and Gynaecology, Faculty of Medicine, Khon Kaen University, Khon Kaen, Thailand; 5 Gender and Women’s Health Unit, Centre for Health Equity, School of Population and Global Health, University of Melbourne, Melbourne, VIC Australia; 6 UNDP/UNFPA/UNICEF/WHO/World Bank Special Programme of Research, Development and Research Training in Human Reproduction (HRP), Department of Sexual and Reproductive Health and Research, World Health Organization, Avenue Appia 20, 1202 Geneva, Switzerland; 7 Department of Anaesthesiology, Faculty of Medicine, Khon Kaen University, Khon Kaen, Thailand; 8 Independent Anaesthetist, Yangon, Myanmar

**Keywords:** Fentanly, Labour pain, Scoping review

## Abstract

**Background:**

Labour pain has been identified as an important reason for women to prefer caesarean section (CS). Fentanyl is one of the short acting opioids recommended by World Health Organization for pain relief during labour. This study aimed to identify and describe the available evidence on the use of fentanyl (monotherapy) for labour pain management by any routes of administration or regime.

**Methods:**

We included the records published until 31 December 2021 which reported administration of fentanyl to women with normal labour for labour pain relief. Data were extracted by one reviewer and checked by another reviewer using a standardised agreement form. We mapped and presented data descriptively in figure and tabular format.

**Results:**

We included 51 records from 49 studies in our scoping review. The studies were conducted in 12 countries, mostly high-income countries. The study designs of the 51 included records were varied as follows: 38 (74.5%) experimental studies (35 randomised controlled trials and three quasi-experimental studies), and 12 (23.5%) observational studies (five retrospective cohort studies, four prospective cohort studies, two retrospective descriptive studies, and one descriptive study) and one qualitative study. Of the included records, six used intranasal fentanyl, five used subcutaneous fentanyl, 18 (35.3%) used intravenous fentanyl, 18 (35.3%) used intrathecal fentanyl, and nine used epidural fentanyl. Many records compared fentanyl with another analgesic agent while five records (9.8%) had no comparison group and seven records (13.7%) compared with no analgesia group. The doses of fentanyl varied by routes, study and the requirement depended on the women. Pain assessment was the most frequent outcome measure presented in the records (78.4%). Only nine records (17.6%) investigated women’s satisfaction about labour pain relief using fentanyl and seven records (13.7%) reported the effect of fentanyl on breastfeeding. The most common reported neonatal outcomes were foetal heart rate (33 records, 64.7%) and Apgar score (32 records, 62.7%).

**Conclusion:**

There is limited primary evidence especially randomised controlled trials to evaluate the effectiveness and harms of different routes of fentanyl in low- or middle-income countries. There is a need for high-quality research to establish the most effective route of fentanyl and associated effects for evidence-based international guidelines.

**Supplementary Information:**

The online version contains supplementary material available at 10.1186/s12884-022-05169-x.

## Background

### Description of the condition under consideration

Over the last three decades, caesarean section (CS) rates have been increasing in many countries to unprecedented levels. Increasing CS rates are a public health concern due to maternal and perinatal risks, cost issues, healthcare efficiency, and inequities [1–3]. Globally, the CS rates nearly doubled from 12% in 2010 to 21% in 2015 and are expected to continue increasing during this decade in the absence of global effective interventions to revert the trend. [4]. The CS rate varied with lowest of 0.6% to highest of 58.1% across countries [5]. Caesarean section can save the lives of women and babies if clinically indicated, while unnecessary CS can create surgical risks rather than benefits [6–8]. Women who had undergone a CS are at higher risk of complications in the following pregnancy such us placenta accrete, placenta previa, uterine rupture or adhesions [9–16]. Babies could also have adverse effects of CS such as stillbirth and preterm birth, necessity of intensive care, low birth weight [12, 17]. Furthermore, there is emerging evidence that babies born by CS may be at higher risk of allergy, atopy, asthma or obesity [17].

Increased CS rate have been influenced by many factors, both medical and non-medical. Medical factors include the increase in childbearing age, maternal body mass index, and clinical conditions such as presence of previous scar, foetal distress, etc. Non-medical factors have been also documented such as maternal request, financial incentives, and lack of supervision and regulations were contributed to increasing CS rate [18–20].

Pain is a common occurrence for women during labour and birth. However, not all women have the same experience. Some women tolerate labour pain well, while others suffer seriously from it. Labour pain has been identified as an important reason for women to request CS [21]. In China, pain-free vaginal childbirth is promoted in response to a dramatic increase in CS rate due to maternal request [22, 23]. Furthermore, many countries provide analgesia during labour and vaginal birth [23–26]. The World Health Organization (WHO) recommends the epidural and parenteral opioid analgesia, such as fentanyl, diamorphine and pethidine, for healthy pregnant women requesting pain relief during labour [27]. However, the provision of epidural analgesia requires skilled healthcare providers and continuous monitoring, and is not widely available. Moreover, there are a number of conditions where administration of epidural analgesia is contraindicated (maternal coagulopathy, infection near needle insertion site, active maternal haemorrhage, maternal septicaemia) [28]. Thus, comprehensive mapping of the recommended alternatives such as the parenteral opioid analgesia is crucial to improved understanding and optimize options and research of pain relief to women in labour.

### Description of the intervention

Parenteral opioid analgesia is a well-established method of relieving labour pain [29, 30]. Pethidine has long been used to manage labour pain and is one of the most commonly used opioids. However, its active metabolite called norpethidine can have adverse effects to both women and baby [31–33].

Fentanyl is a short acting and potent opioid and considered as a good option for labour pain relief [34]. As fentanyl has no active metabolites and produces less sedation, nausea and vomiting, it is useful for women in early active labour and for women with contraindications to epidural analgesia [35]. Although the effectiveness, safety and efficacy of various routes and dosages of fentanyl on labour pain have been documented [36–39], synthesizing and mapping all the available evidence is most likely to provide essential information to the healthcare providers and women in pain management during labour. Fentanyl can be administered via intranasal, subcutaneous, intravenous, intramuscular, intrathecal, or epidural routes to reduce labour pain [37–40]. Some of these routes are straightforward to manage, while others require close monitoring by healthcare providers. It can be administered alone or in combination with another drug [12, 15–17, 19–24].

### How the intervention might work

Fentanyl acts rapidly on spinal cord and brain receptors, blocking signal from the uterus and vagina as pain. The potential adverse effects of fentanyl include a slowed heart rate, nausea and vomiting. Contraindications to fentanyl include hypotension, allergy to fentanyl, liver or respiratory diseases [35, 41].

## Why it is important to do this review?

There are systematic reviews on effectiveness of parenteral opioids for labour pain management, but none specifically on fentanyl [29, 30]. The purpose of this scoping review is to gather, organise and map the available evidence on the use of fentanyl in the management of labour in a systematic manner in order to identify significant research areas and greater depth in subsequent systematic reviews.

## Objectives

To identify the research conducted using fentanyl (monotherapy) for analgesia during labour and systematically describe and map the studies, designs, routes of administration, regimens used, comparators and outcomes studied to date.

## Methods

A protocol of this scoping review was registered at the Open Science Framework (Registration DOI—10.17605/OSF.IO/WCRZ7). This scoping review is reported according to the Preferred Reporting Items for Systematic reviews and Meta-Analyses extension for Scoping Reviews (PRISMA-ScR) statement (Supplementary table S1).

### Criteria for considering studies for the review

#### Type of studies

We included both qualitative and quantitative studies regardless of publication year and language, including descriptive study, interrupted time series, randomised controlled trials (RCTs), quasi-RCTs, prospective and retrospective cohort studies, and before and after studies. This review did not include narrative literatures, case reports, and not original research. Studies that were originally published in a language other than English were translated into English using Google Translate.

#### Types of participants

Women with normal pregnancy either singleton or multiple pregnancies in any age group who needed pain relief during labour. We excluded women with any obstetric or medical complications.

#### Types of interventions

We were particularly interested in the administration of fentanyl to women in labour for pain relief. We excluded administration of fentanyl as an analgesic agent to undergo CS or for other analgesic effect during surgery. We included studies in which fentanyl (monotherapy) was administered for pain relief at least in one trial arm during vaginal labour. Otherwise, we considered as ‘wrong intervention’ and excluded the studies.

#### Types of outcome measures

We included all outcomes reported in the included records evaluating the effects of fentanyl for labour pain management. The outcome measures included visual analogue scale (VAS) on pain, maternal vital signs, duration of analgesia, duration of labour, maternal and perinatal outcomes and adverse events, breastfeeding problems, and maternal satisfaction.

### Search strategy

To identify the potentially relevant evidence, search strategies were developed using the synonyms of labour and fentanyl terms. Boolean operators and medical subject headings (MeSH) were used to develop a search strategy for each electronic database. The search was conducted through the utilization of the following electronic databases: Cochrane Central Register of Controlled Trials (CENTRAL), PubMed, CINAHL, Scopus, Web of Science (ISI), Ovid (Medline), and Open Grey. We also identified trial registrations by searching in WHO International Clinical Trials Registry Platform and ClinicalTrials.gov databases. The search strategies for each database are available in Supplementary table S2. The search encompassed all potentially relevant published and unpublished literature that had been disseminated until 31 December 2021.

Additionally, we searched the reference lists of the retrieved articles and included articles that met our pre-defined criteria and presented sufficient information.

### Selection process

Mendeley software was used to identify and merge search results [42]. Rayyan software was used to screen and select studies [43]. Two researchers independently screened the title and abstracts of the retrieved citations and selected potentially relevant studies for full-text reading (KLS and WYHW). Similarly, two researchers independently assessed the full text of the selected studies using pre-defined selection criteria (KLS, KK, CD, and MS). Discrepancies were resolved through discussion, and a third reviewer was consulted if required. For potential studies that we could not find published full reports, we contacted the corresponding investigators for more information.

### Data collection

A data extraction form specifically designed for this review was prepared in Microsoft Excel. The form was tested and revisions were made as necessary following a discussion among researchers. Data were extracted by one reviewer (KLS) using a standardised agreement data extraction form and counter checked by another reviewer (KK, CD, or MS). We extracted the following information: authors, publication year, citation, funding source, objectives, study design, study setting, sample characteristics (e.g. age group, labour stage), intervention characteristics (route of administration, regime, sample size), comparator characteristics (route, dose, sample size), data collection procedure, and conclusions.

### Data analyses and data visualization

We mapped the extracted information in tabular or figure form and present a descriptive summary of the relevant information in the included records using frequency and proportion for categorical variables, and median and interquartile range (IQR) for the continuous variables. The figure was drawn in the Microsoft Excel. We mapped and reported the results as follows:

1. Description of included records: summary characteristics (country where the study was conducted, year, sample size) of the included records in tabular format are presented.
2. Description of fentanyl and comparators: summary descriptions of the route of fentanyl and its comparator and study design used in the records are provided in this section.
3. Description of outcomes reported in included records.

## Results

### Results of the search

**Fig. 1:**
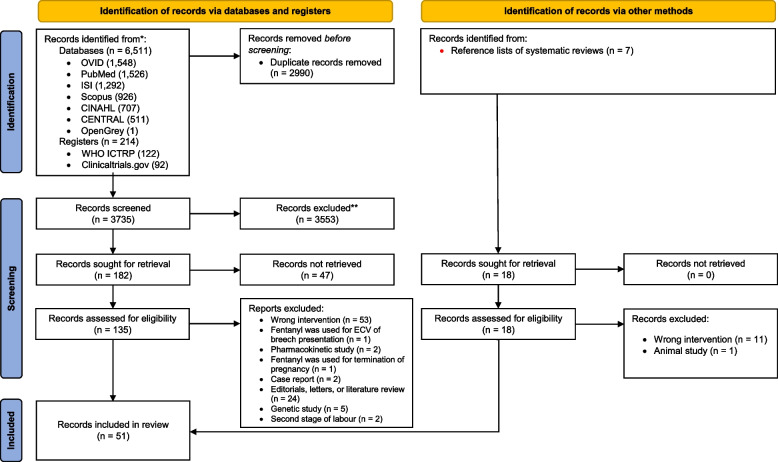
PRISMA flow diagram

We identified a total of 6743 records, consisting of 6725 records from electronic database searches, and 18 records from other sources. After removing 2990 duplicates from the electronic databases, the titles and abstracts of 3735 records were reviewed. We excluded 3553 irrelevant records and assessed 153 records at full-text level. 102 records were excluded at the full text stage and the reasons for their exclusion are listed Supplementary table S4. We therefore included 51 records in this scoping review (Supplementary table S3). Of the included records, three are from different phases of one study but presented in different designs and outcomes [44–46]. Figure 1 illustrates PRISMA flow diagram on the searching and selection processes.

### Characteristics of included records

**Fig. 2:**
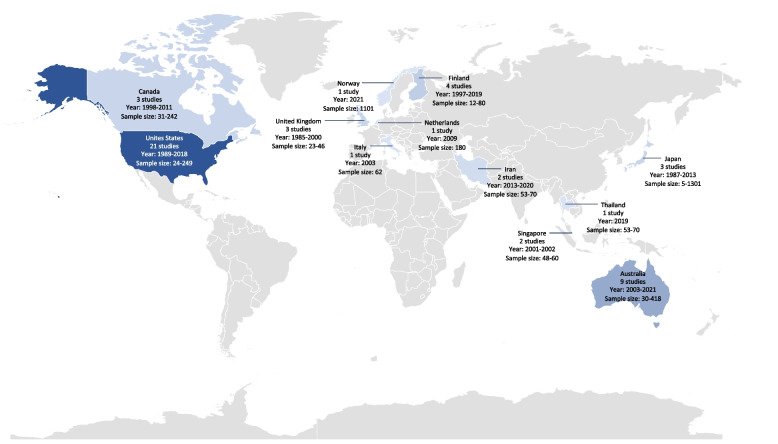
Countries of included records

Of the 51 included records (Table 1 and Supplementary table S5) with 7211 pregnant women, 21 (41.2%, with 1473 participants) records were conducted in United States of America (USA), and nine records (17.6%, with 1285 participants) from Australia. The reported number of pregnant women included in the records ranged from 5 to 1301 pregnant women with a median of 60 pregnant women (IQR: 43–104). The records were published from 1985 to 2021 (Table 1 and Fig. 2).

**Table 1:** Main characteristics of 51 records included in the scoping review

Characteristic	N of records (*N* = 51)	Number of women (*N* = 7211)
	**n (%)**	
Economic category (UN)		
High-income country	48 (94.1)	6996
Low- and middle-income country	3 (5.9)	215
Geographical region		
Asia	8 (15.7)	2469
Europe	10 (19.6)	1613
North America	24 (47.1)	1844
Oceania	9 (17.6)	1285
Study design		
RCT	35 (68.6)	2319
Quasi-experimental	3 (5.9)	286
Observational	12 (23.5)	4490
Qualitative	1 (1.9)	116
Year of publication		
Before 2000	18 (35.3)	1120
2000 to 2009	13 (25.5)	816
2010–2021	20 (39.2)	5275
Sample size		
< 50	17 (33.3)	532
50–99	20 (39.2)	1364
100–500	11 (21.6)	2073
> 500	3 (5.9)	3242

**Fig. 3:**
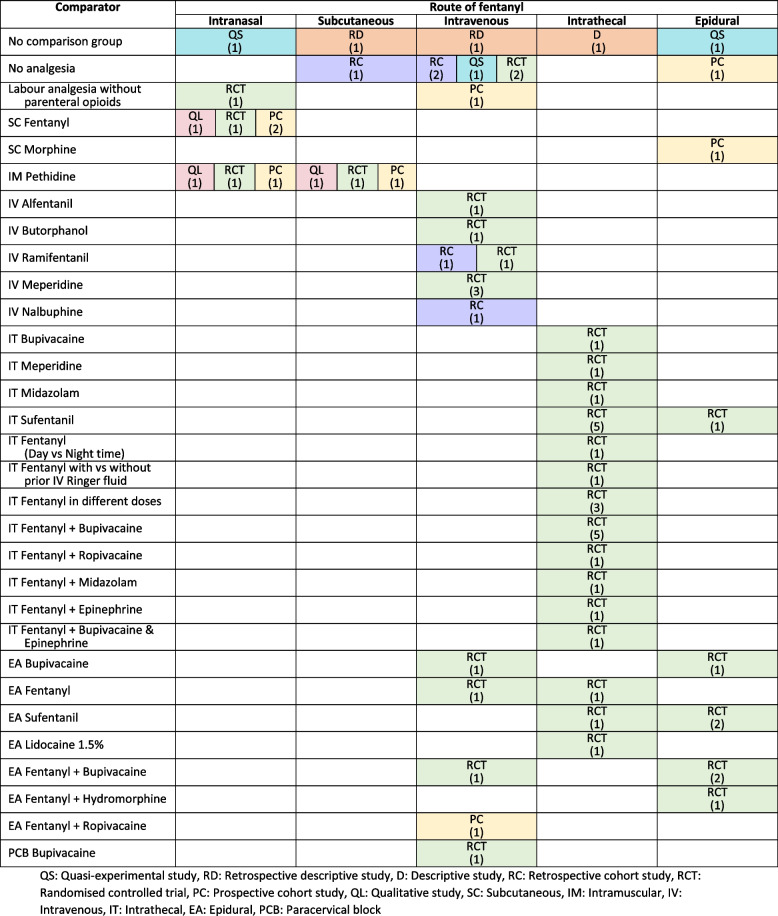
Route of fentanyl mapped by its comparator and study design in 51 included records QS: Quasi-experimental study, RD: Retrospective descriptive study, D: Descriptive study, RC: Retrospective cohort study, RCT: Randomised controlled trial, PC: Prospective cohort study, QL: Qualitative study, SC: Subcutaneous, IM: Intramuscular, IV: Intravenous, IT: Intrathecal, EA: Epidural, PCB: Paracervical block

The study designs of 51 included records were as follows: 38 (74.5%) experimental studies (35 randomised controlled trials and three quasi-experimental studies), and 12 (23.5%) observational studies (five retrospective cohort studies, four prospective cohort studies, 2 retrospective descriptive studies, one descriptive study), and one qualitative study (Fig. 3 and Supplementary table S5).

### Description of fentanyl and comparators

The administration routes of fentanyl described in the included records varied. Six records used intranasal fentanyl, five records used subcutaneous fentanyl, 18 records (35.3%) used intravenous fentanyl, 18 records (35.3%) used intrathecal fentanyl, and nine records used epidural fentanyl. Figure 3 presented the route of fentanyl administration in the included records mapped by its comparison and study design. Many records (39, 76.5%) compared fentanyl to another analgesic agent while five records (9.8%) had no comparison group and seven records (13.7%) compared fentanyl to no analgesia group.

The regimes of fentanyl varied according to the route, the studies, and the needs of the women. Table 2 described the loading and maintenance doses of fentanyl by route of administration. For intranasal fentanyl (*n* = 6), the loading dose ranged between 54 µg and 250 µg with the maximum hourly dose of 600 µg and the maximum total dose was 1200 µg. Subcutaneous fentanyl (*n* = 5) started with 200 µg loading dose and additional dose of same drug as requested by the women after one hour up to a maximum dose of 650 µg. The loading dose for intravenous fentanyl (*n* = 18) ranged from 25 to 100 μg and the maintenance dose varied by study. For intrathecal fentanyl (*n* = 18), the loading dose ranged from 5 to 75 μg and the maintenance dose could be the same drug or other drugs. The loading dose of epidural fentanyl (*n* = 9) ranged from 20 to 125 µg and the maintenance was by other drugs.

**Table 2:** Regimens of fentanyl used in included records by route of administration

Route of Fentanyl	Number of records	Loading Dose (range)	Maintenance Dose
Intranasal Fentanyl	6	54–250 µg	The maximum hourly dose was 600 µg, with a maximum total dose of 1200 µg
Subcutaneous Fentanyl	5	200 µg	After 1 h, additional 50 µg doses could be administered every 15 min, as requested, up to a maximum of 650 µg
Intravenous Fentanyl	18	25–100 μg	- Same dose every 1–2 h - IV-PCA pump 20 μg, lockout interval 3–6 min. The maximum dose of 240 μg per hour, or four-hour limit of 1000–1500 μg in total - Additional 50 μg was given and repeated every 5–10 min until the patient reported adequate pain relief
Intrathecal Fentanyl	21	5–75 μg	- If analgesia is inadequate after 15 min, a second dose of same study solution was injected - Other drugs
Epidural Fentanyl	9	20–125 μg	Other drugs

### Description of outcomes reported in included records

The included records reported a total of 51 unique outcomes, including 35 maternal outcomes (68.6%) and 16 neonatal outcomes (31.4%). Table 3 provides the frequency of category outcomes, which are summarised in the following sections.

**Table 3:** Outcomes reported in included records

Outcomes	Experimental studies^1^(*n* = 38)		Observa-tional studies^2^(*n* = 12)		Qualitative studies(*n* = 1)		All study designs(*n* = 51)	
**Maternal outcomes**								
**Labour pain**								
- Pain score	35	(92.1)	5	(41.7)	0	(0.0)	40	(78.4)
**Maternal assessment**								
- Blood pressure	28	(73.7)	4	(33.3)	0	(0.0)	32	(62.7)
- Maternal Heart Rate	19	(23.7)	4	(33.3)	0	(0.0)	23	(45.1)
- Respiratory Rate	17	(44.7)	3	(25.0)	0	(0.0)	20	(39.2)
- Motor block	15	(39.5)	1	(8.3)	0	(0.0)	16	(31.4)
- SPO2	9	(23.7)	3	(25.0)	0	(0.0)	12	(23.5)
- Sensory level	10	(26.3)	0	(0.0)	0	(0.0)	10	(19.6)
- Vital signs	2	(5.3)	0	(0.0)	0	(0.0)	2	(3.9)
- Fever	1	(2.6)	0	(0.0)	0	(0.0)	1	(2.0)
**Delivery outcomes**								
- Mode of delivery	20	(52.6)	9	(75.0)	0	(0.0)	29	(56.9)
- Duration of labour	16	(42.1)	5	(41.7)	0	(0.0)	21	(41.2)
- Induction of labour	7	(18.4)	8	(66.7)	0	(0.0)	15	(29.4)
- Duration/Frequency of contraction	7	(18.4)	1	(8.3)	0	(0.0)	8	(15.7)
- Duration of postpartum hospital stay	1	(2.6)	3	(25.0)	0	(0.0)	4	(7.8)
**Analgesia**								
- Duration of analgesia	19	(23.7)	4	(33.3)	0	(0.0)	23	(45.1)
- Plasma/CSF fentanyl concentration	6	(15.8)	2	(16.7)	0	(0.0)	8	(15.7)
- Request for additional analgesia	5	(13.2)	1	(8.3)	0	(0.0)	6	(11.8)
- Time to request additional analgesia	4	(10.5)	0	(0.0)	0	(0.0)	4	(7.8)
**Adverse effects**								
- Nausea	25	(65.8)	3	(25.0)	0	(0.0)	28	(54.9)
- Vomiting	25	(65.8)	3	(25.0)	0	(0.0)	28	(54.9)
- Pruritus	25	(65.8)	1	(8.3)	0	(0.0)	26	(51.0)
- Sedation	21	(55.3)	3	(25.0)	0	(0.0)	24	(47.1)
- Headache	5	(13.2)	0	(0.0)	0	(0.0)	5	(9.8)
- Shivering	4	(10.5)	0	(0.0)	0	(0.0)	4	(7.8)
- Neurological symptoms (numbness, leg weakness)	4	(10.5)	0	(0.0)	0	(0.0)	4	(7.8)
- Subjective maternal adverse effects	2	(5.3)	0	(0.0)	0	(0.0)	2	(3.9)
- Nasal irritation	1	(2.6)	0	(0.0)	0	(0.0)	1	(2.0)
- Post-partum haemorrhage	0	(0.0)	1	(8.3)	0	(0.0)	1	(2.0)
- Use of bag mask ventilation	0	(0.0)	1	(8.3)	0	(0.0)	1	(2.0)
- Maternal intubation	0	(0.0)	1	(8.3)	0	(0.0)	1	(2.0)
- Maternal naloxone	0	(0.0)	1	(8.3)	0	(0.0)	1	(2.0)
- Maternal SpO2 < 90	0	(0.0)	1	(8.3)	0	(0.0)	1	(2.0)
**Maternal stress**								
- Norepinephrine and Epinephrine concentration in maternal blood	1	(2.6)	0	(0.0)	0	(0.0)	1	(2.0)
**Breastfeeding status/problems**								
- Breastfeeding status/problems	2	(5.3)	4	(33.3)	1	(100)	7	(13.7)
**Satisfaction**								
- Satisfaction on pain relief	7	(18.4)	1	(8.3)	1	(100)	9	(17.6)
**Neonatal outcomes**								
- Foetal Heart Rate	31	(81.6)	2	(16.7)	0	(0.0)	33	(64.7)
- Apgar score	23	(60.5)	9	(75.0)	0	(0.0)	32	(62.7)
- Cord blood gases	14	(36.8)	5	(41.7)	0	(0.0)	19	(37.3)
- Birthweight	9	(23.7)	7	(58.3)	0	(0.0)	16	(31.4)
- Naloxone requirement	9	(23.7)	5	(41.7)	0	(0.0)	14	(27.5)
- Neurologic & Adaptive Capacity Score	10	(26.3)	0	(0.0)	0	(0.0)	10	(19.6)
- Resuscitation efforts	4	(10.5)	4	(33.3)	0	(0.0)	8	(15.7)
- NICU/Nursery admission	1	(2.6)	4	(33.3)	0	(0.0)	5	(9.8)
- Neonatal fever	2	(5.3)	1	(8.3)	0	(0.0)	3	(5.9)
- Neonatal SPO2	3	(7.9)	0	(0.0)	0	(0.0)	3	(5.9)
- Time to establish breathing	1	(2.6)	2	(16.7)	0	(0.0)	3	(5.9)
- Congenital anomalies	1	(2.6)	1	(8.3)	0	(0.0)	2	(3.9)
- Fetal body movement	2	(5.3)	0	(0.0)	0	(0.0)	2	(3.9)
- Requirement of CPAP	1	(2.6)	1	(8.3)	0	(0.0)	2	(3.9)
- Skin cyanosis	1	(2.6)	0	(0.0)	0	(0.0)	1	(2.0)
- Requirement of Epinephrine	0	(0.0)	1	(8.3)	0	(0.0)	1	(2.0)

^1^ Experimental studies: randomised controlled trials and quasi-experimental study

^2^ Observational studies: prospective cohort study, retrospective cohort study, and descriptive studies, retrospective descriptive study, qualitative study

### Maternal outcomes

Across all records, a total of 35 different maternal outcomes were reported. Maternal outcomes were categorised into eight domains (Table 3). Among eight domains, pain assessment was the most reported domain; it was measured in 40 records (78.4%). Pain assessment was reported in almost all experimental studies (35 records, 92%) while it was reported in five records (41.7%) of observational studies. For maternal assessment outcomes, maternal blood pressure (32 records, 62.7%), maternal heart rate (23 records, 45%), respiratory rate (20 records, 39.2%), and motor block (16 records, 31.4%) were the most reported outcome measures. Mode of birth (29 records, 56.9%), duration of labour (21 records, 41.2%), duration of analgesia (23 records, 45.1%), and maternal adverse effects such as nausea (28 records, 54.9%), vomiting (28 records, 54.9%), pruritus (26 records, 51%), and sedation (24 records, 47%) were also reported as maternal outcomes. Only seven records (13.7%) reported issues with breastfeeding and nine records (17.6%) reported maternal satisfaction about pain relief after using fentanyl. One qualitative study narratively reported issues with breastfeeding and maternal satisfaction on pain relief.

### Neonatal outcomes

Regarding neonatal outcomes, a total of 16 outcomes were reported across 51 records. The most reported outcomes were foetal heart rate (33 records, 64.7%) and Apgar score (32 records, 62.7%). Other neonatal outcomes included, cord blood gases, birthweight, and naloxone requirement reported in 19, 16, and 14 (37.3%, 31.4%, and 27.5%) records.

## Discussion

### Summary of evidence

This scoping review provides a summary of the available evidence regarding the use of fentanyl by its routes, doses, and outcomes in studies involving healthy women in active labour. Most included records were randomised controlled trials comparing different doses or different routes of same drugs, or other drugs. Most common reported maternal reported outcomes were pain assessment, maternal blood pressure and heart rate, mode of delivery, duration of analgesia, adverse effects (nausea, vomiting, pruritus, and sedation). Most common neonatal reported outcomes were foetal heart rate and Apgar score.

Most of the studies included in this scoping review were conducted in high income countries, while there was limited research conducted in low- or middle-income countries. WHO recommends that all healthy pregnant women are offered pain relief during labour based on their preferences, and ideally with a choice of pain management options [47]. Furthermore, satisfactory pain management during labour could reduce the caesarean section rate because labour pain was documented as major reason for requesting caesarean section by mothers. Therefore, the availability of options for management of labour pain is recommended in many countries [23, 26, 35]. This scoping review identified few studies from developing countries probably due to the fact that availability of pain relief during labour is uncommon because of limited resources and access to healthcare, which remained the primary issue [27].

There were many different drug comparisons described in the included studies, and most comparisons were conducted in a small number of RCTs, thus complicating future systematic reviews of intervention effectiveness. Most studies included in this review administered fentanyl by intrathecal or intravenous routes. Intrathecal method is currently the most common pain relief method for labour pain management because of its excellent analgesia action while allowing mother to awake and cooperative during the delivery process with little maternal and neonatal adverse effects. Intravenous administration of fentanyl is also common because it is easy to administer and patients can administer themselves (patient-controlled analgesia). However, parenteral opioid can readily across the placenta and there is concern with the risks to the fetus such as respiratory depression [34, 48].

Although many studies used the visual analogue scale, only few studies explored the woman’s satisfaction about labour pain relief using fentanyl. The importance of improving quality of care as a pathway to achieving effective universal health coverage under Sustainable Development Goal 3: ensuring healthy lives and promoting well-being for all at all ages has been highlighted [49]. Since WHO emphasizes the crucial contribution of experience of and satisfaction with care to effectively achieving quality of care for pregnant women and their newborns [50], we suggest that mother’s satisfaction with pain relief is systematically included as an outcome in future studies. Many studies included maternal adverse effects, and neonatal conditions as outcome measures. Fentanyl given during labour may depress the neonatal reflexes associated with infant's suckling which make difficulties in early exclusive breastfeeding [51, 52]. However, limited research investigated the effect of fentanyl on breastfeeding and most of these studies used observational study design.

### Strengths and limitations

This is, as far as we are aware, the first scoping review to map the available evidence of fentanyl for labour pain management at a global scale. We included all the settings, countries, fentanyl routes and regimes, outcome measures and there were no language restrictions in our review. There were some challenges and limitations in our scoping review. Due to limited time and resources, data extraction was done by a single reviewer instead of by two reviewers independently. However, we tried to minimize errors in data extraction by conducting a counter-checked by another reviewer. In addition, at least two reviewers performed the screening. As scoping reviews aim to provide a comprehensive overview of the literature on a specific topic, neither risk of bias nor certainty of evidence assessment or grading is required and thus was not performed. 

### Implications for future research

This review identified the available evidence on the use of fentanyl in various routes for labour pain management. There is limited primary evidence especially randomized controlled trials to evaluate the effectiveness and harms of different routes of fentanyl in developing countries.

## Conclusion

This scoping review identified 51 records on the use of fentanyl in labour pain management. There are few studies reported from developing countries. Although clinical outcomes are reported in all studies, few studies reported maternal satisfaction on the pain relief by using fentanyl during labour. There is limited primary evidence especially randomized controlled trials to evaluate the effectiveness and harms of different routes of fentanyl in developing countries.

## Supplementary Information

**Additional file 1:**

## Data Availability

The data used in this current study is available from the corresponding author on reasonable request.
