# Supplementary material for: Fentanyl for labour pain management: a scoping review
Source: BMC Pregnancy Childbirth. 2022 Nov 17;22:846. doi: 10.1186/s12884-022-05169-x (PMC9670642; doi:10.1186/s12884-022-05169-x)
Supplement: Supplementary file 1 — Additional file 1: [file 12884_2022_5169_MOESM1_ESM.docx]

**Supplementary table S1.** Preferred Reporting Items for Systematic reviews and Meta-Analyses extension for Scoping Reviews (PRISMA-ScR) Checklist

| **SECTION** | **ITEM** | **PRISMA-ScR CHECKLIST ITEM** | **REPORTED ON PAGE #** |
| --- | --- | --- | --- |
| **TITLE** | | | |
| Title | 1 | Identify the report as a scoping review. | 1 |
| **ABSTRACT** | | | |
| Structured summary | 2 | Provide a structured summary that includes (as applicable): background, objectives, eligibility criteria, sources of evidence, charting methods, results, and conclusions that relate to the review questions and objectives. | 2 |
| **INTRODUCTION** | | | |
| Rationale | 3 | Describe the rationale for the review in the context of what is already known. Explain why the review questions/objectives lend themselves to a scoping review approach. | 5 |
| Objectives | 4 | Provide an explicit statement of the questions and objectives being addressed with reference to their key elements (e.g., population or participants, concepts, and context) or other relevant key elements used to conceptualize the review questions and/or objectives. | 6 |
| **METHODS** | | | |
| Protocol and registration | 5 | Indicate whether a review protocol exists; state if and where it can be accessed (e.g., a Web address); and if available, provide registration information, including the registration number. | 6 |
| Eligibility criteria | 6 | Specify characteristics of the sources of evidence used as eligibility criteria (e.g., years considered, language, and publication status), and provide a rationale. | 6 |
| Information sources* | 7 | Describe all information sources in the search (e.g., databases with dates of coverage and contact with authors to identify additional sources), as well as the date the most recent search was executed. | 7 |
| Search | 8 | Present the full electronic search strategy for at least 1 database, including any limits used, such that it could be repeated. | Supplementary table S2 |
| Selection of sources of evidence† | 9 | State the process for selecting sources of evidence (i.e., screening and eligibility) included in the scoping review. | 7 |
| Data charting process‡ | 10 | Describe the methods of charting data from the included sources of evidence (e.g., calibrated forms or forms that have been tested by the team before their use, and whether data charting was done independently or in duplicate) and any processes for obtaining and confirming data from investigators. | 8 |
| Data items | 11 | List and define all variables for which data were sought and any assumptions and simplifications made. | 8 |
| Critical appraisal of individual sources of evidence§ | 12 | If done, provide a rationale for conducting a critical appraisal of included sources of evidence; describe the methods used and how this information was used in any data synthesis (if appropriate). | NA |
| Synthesis of results | 13 | Describe the methods of handling and summarizing the data that were charted. | 8 |
| **RESULTS** | | | |
| Selection of sources of evidence | 14 | Give numbers of sources of evidence screened, assessed for eligibility, and included in the review, with reasons for exclusions at each stage, ideally using a flow diagram. | 8 |
| Characteristics of sources of evidence | 15 | For each source of evidence, present characteristics for which data were charted and provide the citations. | 9 |
| Critical appraisal within sources of evidence | 16 | If done, present data on critical appraisal of included sources of evidence (see item 12). | NA |
| Results of individual sources of evidence | 17 | For each included source of evidence, present the relevant data that were charted that relate to the review questions and objectives. | Supplementary table S5 |
| Synthesis of results | 18 | Summarize and/or present the charting results as they relate to the review questions and objectives. | 9 |
| **DISCUSSION** | | | |
| Summary of evidence | 19 | Summarize the main results (including an overview of concepts, themes, and types of evidence available), link to the review questions and objectives, and consider the relevance to key groups. | 18 |
| Limitations | 20 | Discuss the limitations of the scoping review process. | 19 |
| Conclusions | 21 | Provide a general interpretation of the results with respect to the review questions and objectives, as well as potential implications and/or next steps. | 20 |
| **FUNDING** | | | |
| Funding | 22 | Describe sources of funding for the included sources of evidence, as well as sources of funding for the scoping review. Describe the role of the funders of the scoping review. | 20 |

JBI = Joanna Briggs Institute; PRISMA-ScR = Preferred Reporting Items for Systematic reviews and Meta-Analyses extension for Scoping Reviews.

* Where *sources of evidence* (see second footnote) are compiled from, such as bibliographic databases, social media platforms, and Web sites.

† A more inclusive/heterogeneous term used to account for the different types of evidence or data sources (e.g., quantitative and/or qualitative research, expert opinion, and policy documents) that may be eligible in a scoping review as opposed to only studies. This is not to be confused with *information sources* (see first footnote).

‡ The frameworks by Arksey and O’Malley (6) and Levac and colleagues (7) and the JBI guidance (4, 5) refer to the process of data extraction in a scoping review as data charting*.*

§ The process of systematically examining research evidence to assess its validity, results, and relevance before using it to inform a decision. This term is used for items 12 and 19 instead of "risk of bias" (which is more applicable to systematic reviews of interventions) to include and acknowledge the various sources of evidence that may be used in a scoping review (e.g., quantitative and/or qualitative research, expert opinion, and policy document).

*From:* Tricco AC, Lillie E, Zarin W, O'Brien KK, Colquhoun H, Levac D, et al. PRISMA Extension for Scoping Reviews (PRISMAScR): Checklist and Explanation. Ann Intern Med. 2018;169:467–473. [doi: 10.7326/M18-0850](http://annals.org/aim/fullarticle/2700389/prisma-extension-scoping-reviews-prisma-scr-checklist-explanation).

**Supplementary table S2.** Search strategy of each database

**CINAHL Database**

| **Search** | **Query** |
| --- | --- |
| S1 | TX "fentanyl" OR "phentanyl" OR "fentanest" OR "fentanyl citrate" OR "oral transmucosal fentanyl citrate" OR "R-4263" OR "R 4263" OR "R4263" OR "sublimaze" OR "duragesic" OR "durogesic" OR "fentora" OR "Abstral" OR "Actiq" OR "Fentora" OR "Onsolis" OR "Duragesic" OR "Sublimaze" OR "Matrifen" OR "Haldid" OR "Instanyl" OR "Lazanda" OR fentan* |
| S2 | TX ( "labor" OR "labour" OR "obstetric" OR "childbirth" OR "child birth" OR deliver* OR parturient* ) N3 "pain" |
| S3 | TX (( "uterine" N2 "contraction" ) OR ( "pressure" N2 "cervix" )) N3 "pain" |
| S4 | S2 OR S3 |
| S5 | S1 AND S4 |
| S6 | S1 AND S4 (April to Dec 2021) |

**Cochrane Central**

| **#** | **Searches** |
| --- | --- |
| #1 | MeSH descriptor: [Fentanyl] explode all trees |
| #2 | "fentanyl" OR "phentanyl" OR "fentanest" OR "fentanyl citrate" OR "oral transmucosal fentanyl citrate" OR "R-4263" OR "R 4263" OR "R4263" OR "sublimaze" OR "duragesic" OR "durogesic" OR "fentora" OR "Abstral" OR "Actiq" OR "Fentora" OR "Onsolis" OR "Duragesic" OR "Sublimaze" OR "Matrifen" OR "Haldid" OR "Instanyl" OR "Lazanda" OR "fentan*" |
| #3 | #1 OR #2 |
| #4 | MeSH descriptor: [Labor, Obstetric] explode all trees |
| #5 | MeSH descriptor: [Pain] explode all trees |
| #6 | #4 AND #5 |
| #7 | MeSH descriptor: [Labor Pain] explode all trees |
| #8 | ("labor" OR "labour" OR "obstetric" OR "childbirth" OR "child birth" OR deliver* OR parturient*) NEAR/3 "pain" |
| #9 | (("uterine" NEAR/2 "contraction") OR ("pressure" NEAR/2 "cervix")) NEAR/3 "pain" |
| #10 | #8 OR #9 |
| #11 | #6 OR #7 OR #10 |
| #12 | #3 AND #11 |
| #13 | #12 in Trials |

**ISI Web of Science**

| **#** | **Searches** |
| --- | --- |
| #1 | ALL = ("fentanyl" OR "phentanyl" OR "fentanest" OR "fentanyl citrate" OR "oral transmucosal fentanyl citrate" OR "R-4263" OR "R 4263" OR "R4263" OR "sublimaze" OR "duragesic" OR "durogesic" OR "fentora" OR "Abstral" OR "Actiq" OR "Fentora" OR "Onsolis" OR "Duragesic" OR "Sublimaze" OR "Matrifen" OR "Haldid" OR "Instanyl" OR "Lazanda" OR "fentan*") |
| #2 | ALL = ( ( "labor" OR "labour" OR "obstetric" OR "childbirth" OR "child birth" OR deliver* OR parturient* ) AND "pain" ) |
| #3 | ALL = ( ( ( "uterine" AND "contraction" ) OR ( "pressure" AND "cervix" ) ) AND "pain" ) |
| #4 | #2 OR #3 |
| #5 | #1 AND #4 |

**Ovid Database**

| **Search** | **Query** |
| --- | --- |
| #1 | exp Fentanyl/ |
| #2 | ("fentanyl" or "phentanyl" or "fentanest" or "fentanyl citrate" or "oral transmucosal fentanyl citrate" or "R-4263" or "R 4263" or "R4263" or "sublimaze" or "duragesic" or "durogesic" or "fentora" or "Abstral" or "Actiq" or "Fentora" or "Onsolis" or "Duragesic" or "Sublimaze" or "Matrifen" or "Haldid" or "Instanyl" or "Lazanda" or "fentan*").af. |
| #3 | 1 OR 2 |
| #4 | exp Labor, Obstetric/ |
| #5 | exp Pain/ |
| #6 | 4 AND 5 |
| #7 | exp Labor Pain/ |
| #8 | (("labor" or "labour" or "obstetric" or "childbirth" or "child birth" or deliver* or parturient*) and "pain").af. |
| #9 | ((("uterine" and "contraction") or ("pressure" and "cervix")) and "pain").af. |
| #10 | 8 OR 9 |
| #11 | 6 OR 7 OR 10 |
| #12 | 3 AND 11 |

**PubMed**

| **Search** | **Query** |
| --- | --- |
| #1 | fentanyl [MH] |
| #2 | "fentanyl" OR "phentanyl" OR "fentanest" OR "fentanyl citrate" OR "oral transmucosal fentanyl citrate" OR "R-4263" OR "R 4263" OR "R4263" OR "sublimaze" OR "duragesic" OR "durogesic" OR "fentora" OR "Abstral" OR "Actiq" OR "Fentora" OR "Onsolis" OR "Duragesic" OR "Sublimaze" OR "Matrifen" OR "Haldid" OR "Instanyl" OR "Lazanda" OR "fentan*" |
| #3 | #1 OR #2 |
| #4 | obstetric labor [MH] AND pain [MH] |
| #5 | labour pain [MH] |
| #6 | ("labor" OR "labour" OR "obstetric" OR "childbirth" OR "child birth" OR deliver* OR parturient*) AND "pain" |
| #7 | (("uterine" AND "contraction") OR ("pressure" AND "cervix")) AND "pain" |
| #8 | #6 OR #7 |
| #9 | #4 OR #5 OR #8 |
| #10 | #3 AND #9 |

**Scopus**

| **#** | **Searches** |
| --- | --- |
| #1 | TITLE-ABS-KEY ( "fentanyl" OR "phentanyl" OR "fentanest" OR ("fentanyl citrate") OR ("oral transmucosal fentanyl citrate") OR "R-4263" OR ("R 4263") OR "R4263" OR "sublimaze" OR "duragesic" OR "durogesic" OR "fentora" OR "Abstral" OR "Actiq" OR "Fentora" OR "Onsolis" OR "Duragesic" OR "Sublimaze" OR "Matrifen" OR "Haldid" OR "Instanyl" OR "Lazanda" OR "fentan*" ) |
| #2 | TITLE-ABS-KEY ( ( "labor" OR "labour" OR "obstetric" OR "childbirth" OR ("child birth") OR deliver* OR parturient* ) W/3 "pain" ) |
| #3 | TITLE-ABS-KEY ( ( ( "uterine" W/2 "contraction" ) OR ( "pressure" W/2 "cervix" ) ) W/3 "pain" ) |
| #4 | #2 OR #3 |
| #5 | #1 AND #4 |

**Open Grey**

( ( "fentanyl" OR "phentanyl" OR "fentanest" OR ( "fentanyl citrate" ) OR ( "oral transmucosal fentanyl citrate" ) OR "R-4263" OR "R 4263" OR "R4263" OR "sublimaze" OR "duragesic" OR "durogesic" OR "fentora" OR "Abstral" OR "Actiq" OR "Fentora" OR "Onsolis" OR "Duragesic" OR "Sublimaze" OR "Matrifen" OR "Haldid" OR "Instanyl" OR "Lazanda" OR "fentan$" ) ) AND ( ( ( ( "labor" OR "labour" OR "obstetric" OR "childbirth" OR ( "child birth" ) OR deliver$ OR parturient$ ) AND "pain" ) ) OR ( ( ( ( "uterine" AND "contraction" ) OR ( "pressure" AND "cervix" ) ) AND "pain" ) ) ) **Supplementary table S3.** References to studies included in this scoping review

|  | Asokumar B, Michael Newman L, McCarthy RJ, Ivankovich AD, Tuman KJ. Intrathecal bupivacaine reduces pruritus and prolongs duration of fentanyl analgesia during labor: A prospective, randomized controlled trial. Anesth Analg. 1998;87(6):1309–15. |
| --- | --- |
|  | Atkinson BD, Truitt LJ, Rayburn WF, Turnbull GL, Dix Christensen H, Wlodaver A. Double-blind comparison of intravenous butorephanol (Stadol) and fentanyl (Sublimaze) for analgesia during labor. Am J Obstet Gynecol. 1994;171(4):993–8. |
|  | Capogna G, Camorcia M, Columb MO. Minimum analgesic doses of fentanyl and sufentanil for epidural analgesia in the first stage of labor. Anesth Analg. 2003;96(4):1178-contents. |
|  | Celeski DC, Heindel L, Haas J, Vacchiano CA. Effect of intrathecal fentanyl dose on the duration of labor analgesia. AANA J. 1999;67(3):239–44. |
|  | Connelly NR PRKVVBS, Dunn S, Connelly NR, Parker RK, Vallurupalli V, Bhopatkar S, et al. Comparison of epidural fentanyl versus epidural sufentanil for analgesia in ambulatory patients in early labor. Anesth Analg. 2000;91(2):374–8. |
|  | Connelly NR, Parker RK, Lucas T, El-Mansouri M, Komanduri V, Nayak P, et al. The influence of a bupivacaine and fentanyl epidural infusion after epidural fentanyl in patients allowed to ambulate in early labor. Anesth Analg. 2001;93(4):1001–5. |
|  | Cascio M, Pygon B, Bernett C, Ramanathan S. Labour analgesia with intrathecal fentanyl decreases maternal stress. Can J Anaesth. 1997;44(6):605–9. |
|  | Douma MR, Verwey RA, Kam-Endtz CE, van der Linden PD, Stienstra R. Obstetric analgesia: a comparison of patient-controlled meperidine, remifentanil, and fentanyl in labour. Br J Anaesth. 2010;104(2):209–15. |
|  | Fleet JA, Jones M, Belan I. Taking the alternative route: Women’s experience of intranasal fentanyl, subcutaneous fentanyl or intramuscular pethidine for labour analgesia. Midwifery. 2017;53(December 2016):15–9. |
|  | Fleet J, Belan I, Jones MJ, Ullah S, Cyna AM. A comparison of fentanyl with pethidine for pain relief during childbirth: a randomised controlled trial. BJOG An Int J Obstet Gynaecol. 2015;122(7):983–92. |
|  | Fleet J. Subcutaneous administration of fentanyl in childbirth: an observational study on the clinical effectiveness of fentanyl for mother and neonate. Women and Birth. 2011;24(February 2009):S36–7. |
|  | Fleet JA, Jones M, Belan I. The influence of intrapartum opioid use on breastfeeding experience at 6 weeks post partum: A secondary analysis. Midwifery. 2017;50(November 2016):106–9. |
|  | Fleet J-A, Belan I, Gordon AL, Cyna AM. Fentanyl concentration in maternal and umbilical cord plasma following intranasal or subcutaneous administration in labour. Int J Obstet Anesth. 2020;42:34–8. |
|  | Fleet J, Sok C, Randall ER, Cyna AM. Subcutaneous fentanyl for labour analgesia: a retrospective case note review. Int J Obstet Anesth. 2021;45(2021):138–41. |
|  | Gaiser RR, Cheek TG, Gutsche BB. Comparison of three different doses of intrathecal fentanyl and sufentanil for labor analgesia. J Clin Anesth. 1998;10(6):488–93. |
|  | Goodman SR, Kim-Lo SH, Ciliberto CF, Ridley DM, Smiley RM. Epinephrine is not a useful addition to intrathecal fentanyl or fentanyl-bupivacaine for labor analgesia. Reg Anesth Pain Med. 2002;27(4):374–9. |
|  | Halpern SH MHBTWCDCBJLR, Blanchard JW, Halpern SH, Muir H, Breen TW, Campbell DC, et al. A multicenter randomized controlled trial comparing patient-controlled epidural with intravenous analgesia for pain relief in labor. Anesth Analg. 2004;99(5):1532–8. |
|  | Herman NL, Choi KC, Affleck PJ, Calicott R, Brackin R, Singhal A, et al. Analgesia, pruritus, and ventilation exhibit a dose-response relationship in parturients receiving intrathecal fentanyl during labor. Anesth Analg. 1999;89(2):378–83. |
|  | Honet JE, Arkoosh VA, Norris MC, Huffnagle HJ, Silverman NS, Leighton BL. Comparison among intrathecal fentanyl, meperidine, and sufentanil for labor analgesia. Anesth Analg. 1992;75(5):734–9. |
|  | Hosokawa Y, Morisaki H, Nakatsuka I, Hashiguchi S, Miyakoshi K, Tanaka M, et al. Retrospective evaluation of intravenous fentanyl patient-controlled analgesia during labor. J Anesth. 2012;26(2):219–24. |
|  | Jahani Shoorab N, Ebrahimzadeh Zagami S, Mirzakhani K, Mazlom SR. The effect of intravenous fentanyl on pain and duration of the active phase of first stage labor. Oman Med J. 2013;28(5):306–10. |
|  | Kerr D, Taylor D, Evans B, Kerr D TD, Evans B. Patient-controlled intranasal fentanyl analgesia: a pilot study to assess practicality and tolerability during childbirth. Int J Obstet Anesth. 2015;24(2):117–23. |
|  | Kokki M, Westeren-Punnonen S, Hautajärvi H, Heinonen S, Mazzei M, Määttä S, et al. Neonatal safety of maternal fentanyl during labour. Br J Anaesth. 2015;115(4):636–8. |
|  | Lim EHL, Sia ATH, Wong K, Tan HM. Addition of bupivacaine 1.25 mg to fentanyl confers no advantage over fentanyl alone for intrathecal analgesia in early labour. Can J Anesth. 2002;49(1):57–61. |
|  | Mahomed K, Wild K, Brown C, Green A. Does fentanyl epidural analgesia affect breastfeeding: A prospective cohort study. Aust N Z J Obstet Gynaecol. 2019;59(6):819–24. |
|  | Miyakoshi K, Tanaka M, Morisaki H, Kim S-H, Hosokawa Y, Matsumoto T, et al. Perinatal outcomes: Intravenous patient-controlled fentanyl versus no analgesia in labor. J Obstet Gynaecol Res. 2013;39(4):783–9. |
|  | Manouchehrian N RSMA, Lakpur Z, Manouchehrian N, Rabiei S, Moradi A, Lakpur Z. Comparison of intrathecal injection of fentanyl and sufentanil on the onset, duration, and quality of analgesia in labor: a randomized, double-blind clinical trial. Anesthesiol pain Med. 2020;10(3):1–7. |
|  | Marwah R, Hassan S, Carvalho JCA, Balki M. Remifentanil versus fentanyl for intravenous patient-controlled labour analgesia: An observational study. Can J Anesth. 2012;59(3):246–54. |
|  | Morley-forster PK, Reid DW, Vandeberghe H. A comparison of patient-controlled analgesia fentanyl and alfentanil for labour analgesia. Can J Anesthe. 2000;113–9. |
|  | Morley-Forster PK, Weberpals J. Neonatal effects of patient-controlled analgesia using fentanyl in labor. Int J Obstet Anesth. 1998;7(2):103–7. |
|  | Murakawa K, Abboud TK, Yanagi T, Sarkis F, Afrasiabi A, Sheikh-ol-Eslam A, et al. Clinical experience of epidural fentanyl for labor pain. J Anesth. 1987;1(1):93–5. |
|  | Nelson KE, Rauch T, Terebuh V, D’Angelo R. A comparison of intrathecal fentanyl and sufentanil for labor analgesia. Anesthesiology. 2002;96(5):1070–3. |
|  | Nelson KE, Houle TT, Eisenach JC. Blood pressure, but not cerebrospinal fluid fentanyl concentration, predicts duration of labor analgesia from spinal fentanyl. Anesthesiology. 2010;112(1):174–80. |
|  | Nguyen T-A, Wang X-F, Wagner K, Izquierdo M, Bolden N. Labor Analgesia when Neuraxial Anesthesia is Relatively Contraindicated : Comparison of Patient-Controlled Fentanyl and Intermittent Nalbuphine Boluses. J Clin Anesth Manag. 2018;1–6. |
|  | Nikkola EM, Ekblad UU, Kero PO, Alihanka JIM, Salonen MAO. Intravenous fentanyl PCA during labour. Can J Anaesth. 1997;44(12):1248–55. |
|  | Nikkola EM, Jahnukainen TJ, Ekblad UU, Kero PO, Salonen MAO. Neonatal Monitoring after Maternal Fentanyl Analgesia in Labor. J Clin Monit Comput. 2001;(16):597–608. |
|  | Oommen H, Oddbjørn Tveit T, Eskedal LT, Myr R, Swanson DM, Vistad I. The association between intrapartum opioid fentanyl and early breastfeeding: A prospective observational study. Acta Obstet Gynecol Scand. 2021 Dec;100(12):2294–302. |
|  | Palmer CM, Maren G Van, Nogami WM, Alves D. Bupivacaine Augments Intrathecal Fentanyl for Labor Analgesia. Anesthesiology. 1999;91(1):206–9. |
|  | Palmer CM, Cork RC, Hays R, Maren G Van, Alves D. The Dose-Response Relation of Intrathecal Fentanyl for Labor Analgesia. Anesthesiology. 1998;88(2). |
|  | Pan PH, Lee S, Harris L, Pan PH, Lee S, Harris L. Chronobiology of subarachnoid fentanyl for labor analgesia. Anesthesiology. 2005;103(3):595–9. |
|  | Parker RK, Connelly NR, Lucas T, Faheem U, Rizvi AS, El-Mansouri M, et al. The addition of hydromorphone to epidural fentanyl does not affect analgesia in early labour. Can J ANAESTHESIA-JOURNAL Can D Anesth. 2002;49(6):600–4. |
|  | Raksakulkiat S. A comparison of meperidine and fentanyl for labor pain reduction in Phramongkutklao hospital. J Med Assoc Thail. 2019;(102):197–202. |
|  | Rayburn WF, Smith CV, Parriott JE, Woods RE. Randomized Comparison of Meperidine and Fentanyl During Labour. Obstet Anesth Dig. 1990;76. |
|  | Rayburn W, Rathke A, Leuschen MP, Chleborad J, Weidner W. Fentanyl citrate analgesia during labor. Am J Obstet Gynecol. 1989;161(1):202–6. |
|  | Reynolds F, O’Sullivan G. Epidural fentanyl and perineal pain in labour. Anaesthesia. 1989;44(October 1988):341–4. |
|  | Shah MK, Sia ATH, Chong JL. The effect of the addition of ropivacaine or bupivacaine upon pruritus induced by intrathecal fentanyl in labour. Anaesthesia. 2000;55(10):1008–13. |
|  | Shannon MT, Ramanathan S. An intravenous fluid bolus is not necessary before administration of intrathecal fentanyl for labor analgesia. J Clin Anesth. 1998;10(6):452–6. |
|  | Smith C V., Rayburn WF, Allen K V., Bane TM, Livezey GT. Influence of intravenous fentanyl on fetal biophysical parameters during labor. J Matern Neonatal Med. 1996;5(2):89–92. |
|  | Tucker AP, Mezzatesta J, Nadeson R, Goodchild CS. Intrathecal midazolam II: Combination with intrathecal fentanyl for labor pain. Anesth Analg. 2004;98(6):1521–7. |
|  | Väänänen A, Kuukasjärvi M, Tekay A, Ahonen J, Vaananen A, Kuukasjarvi M, et al. Spinal and epidural sufentanil and fentanyl in early labour. Acta Anaesthesiol Scand. 2019;63(10):1413–8. |
|  | Vella LM, Willatts DG, Knott C, Lintin DJ, Justins DM, Reynolds F. Epidural fentanyl in labour. Anaesthesia. 1985;40(8):741–7. |

**Supplementary table S4.** References to studies excluded and reasons to exclude from this scoping review

| **References** | **Reason for exclusion** |
| --- | --- |
| Adams J, Frawley J, Steel A, Broom A, Sibbritt D. Use of pharmacological and non-pharmacological labour pain management techniques and their relationship to maternal and infant birth outcomes: examination of a nationally representative sample of 1835 pregnant women. Midwifery. 2015 Apr;31(4):458-63. doi: 10.1016/j.midw.2014.12.012. Epub 2015 Jan 8. PMID: 25649472. | Wrong intervention |
| Ahmet G, Hospital C, Building A. Epidural analgesia for labor pain: what has changed in the last 1 year? Literature review and clinical results our experience in labor analgesia. Ann Clin Anal Med. 2019;10(02):0–3. | Editorials, letters, or literature review |
| Akerman N, Dresner M. The management of breakthrough pain during labour. CNS Drugs. 2009 Aug;23(8):669-79. doi: 10.2165/00023210-200923080-00004. PMID: 19594196. | Editorials, letters, or literature review |
| Anderson D. A review of systemic opioids commonly used for labor pain relief. J Midwifery Womens Health. 2011 May-Jun;56(3):222-39. doi: 10.1111/j.1542-2011.2011.00061.x. Erratum in: J Midwifery Womens Health. 2011 Jul-Aug;56(4):411-8. Erratum in: J Midwifery Womens Health. 2011 Jul;56(4):411-418. PMID: 21535371. | Editorials, letters, or literature review |
| Anwari JS, Ehsan FM, Al-Dar MM. Intravenous patient-controlled analgesia for labor pain. Saudi Med J. 2003 Jun;24(6):691-3. PMID: 12847612. | Case report |
| Beilin Y, Bodian CA, Weiser J, Hossain S, Arnold I, Feierman DE, Martin G, Holzman I. Effect of labor epidural analgesia with and without fentanyl on infant breast-feeding: a prospective, randomized, double-blind study. Anesthesiology. 2005 Dec;103(6):1211-7. doi: 10.1097/00000542-200512000-00016. PMID: 16306734. | Wrong intervention |
| Breen TW, Giesinger CM, Halpern SH. Comparison of epidural lidocaine and fentanyl to intrathecal sufentanil for analgesia in early labour. Int J Obstet Anesth. 1999 Oct;8(4):226-30. doi: 10.1016/s0959-289x(99)80101-0. PMID: 15321115. | Wrong intervention |
| Brimdyr K, Cadwell K, Widström AM, Svensson K, Neumann M, Hart EA, Harrington S, Phillips R. The Association Between Common Labor Drugs and Suckling When Skin-to-Skin During the First Hour After Birth. Birth. 2015 Dec;42(4):319-28. doi: 10.1111/birt.12186. Epub 2015 Oct 13. PMID: 26463582; PMCID: PMC5057303. | Wrong intervention |
| Bruyère M, Mercier FJ. Alternatives à l'analgésie péridurale au cours du travail [Alternative techniques to labour epidural analgesia]. Ann Fr Anesth Reanim. 2005 Nov-Dec;24(11-12):1375-82. French. doi: 10.1016/j.annfar.2005.07.072. Epub 2005 Aug 22. PMID: 16115746. | Editorials, letters, or literature review |
| Campbell DC. Parenteral opioids for labor analgesia. Clin Obstet Gynecol. 2003 Sep;46(3):616-22. doi: 10.1097/00003081-200309000-00014. PMID: 12972743. | Editorials, letters, or literature review |
| Castro C, Windrim R. Patient-controlled analgesia with fentanyl provides effective analgesia for second trimester labour: a randomized controlled study. Can J Anaesth. 2003;50(10). | Fentanyl was used for termination of pregnancy |
| Chau A, Bibbo C, Huang CC, Elterman KG, Cappiello EC, Robinson JN, Tsen LC. Dural Puncture Epidural Technique Improves Labor Analgesia Quality With Fewer Side Effects Compared With Epidural and Combined Spinal Epidural Techniques: A Randomized Clinical Trial. Anesth Analg. 2017 Feb;124(2):560-569. doi: 10.1213/ANE.0000000000001798. PMID: 28067707. | Wrong intervention |
| Cheng CJ, Sia AT, Lim EH, Loke GP, Tan HM (2001) Either sufentanil or fentanyl, in addition to intrathecal bupivacaine, provide satisfactory early labor analgesia. Can J Anesth 48(6):570–574. https://doi.org/10.1007/BF03016834 | Wrong intervention |
| Chen YL, Zeng LR, Guo R (2015) Comparison of the effects of sufentanil and fentanyl on parturients for combined spinal-epidural analgesia during labor. Heilongjiang Med Sci 38(1):32–34. https://doi.org/10.3969/j.issn.1008-0104.2015.01.014 | Wrong intervention |
| Craft JB Jr, Coaldrake LA, Bolan JC, Mondino M, Mazel P, Gilman RM, Shokes LK, Woolf WA. Placental passage and uterine effects of fentanyl. Anesth Analg. 1983 Oct;62(10):894-8. PMID: 6614522. | Animal study |
| Craig MG, Grant EN, Tao W, McIntire DD, Leveno KJ. A randomized control trial of bupivacaine and fentanyl versus fentanyl-only for epidural analgesia during the second stage of labor. Anesthesiology. 2015 Jan;122(1):172-7. doi: 10.1097/ALN.0000000000000454. PMID: 25254902. | Fentanyl was used during second stage of labour |
| Collis RE, Baxandall ML, Srikantharajah ID, Edge G, Kadim MY, Morgan BM. Combined spinal epidural analgesia with ability to walk throughout labour. Lancet. 1993 Mar 20;341(8847):767-8. doi: 10.1016/0140-6736(93)90548-u. Erratum in: Lancet 1993 Apr 17;341(8851):1038. PMID: 8095677. | Wrong intervention |
| Collis RE, Davies DW, Aveling W. Randomised comparison of combined spinal-epidural and standard epidural analgesia in labour. Lancet. 1995 Jun 3;345(8962):1413-6. doi: 10.1016/s0140-6736(95)92602-x. PMID: 7760614. | Wrong intervention |
| Comparative Obstetric Mobile Epidural Trial (COMET) Study Group UK. Effect of low-dose mobile versus traditional epidural techniques on mode of delivery: a randomised controlled trial. Lancet. 2001 Jul 7;358(9275):19-23. doi: 10.1016/S0140-6736(00)05251-X. PMID: 11454372. | Wrong intervention |
| Côrtes CA de F, Sanchez CA, Oliveira AS, Sanchez FM. Labor analgesia: a comparative study between combined spinal-epidural anesthesia versus continuous epidural anesthesia. Rev Bras Anestesiol. 2007 Feb;57(1):39–51. | Wrong intervention |
| DeBalli P, Breen TW. Intrathecal opioids for combined spinal-epidural analgesia during labour. CNS Drugs. 2003;17(12):889-904. doi: 10.2165/00023210-200317120-00003. PMID: 12962528. | Editorials, letters, or literature review |
| Easther RG, Downey K, Watts N, Ye XY, Carvalho JCA. Quality of labor epidural analgesia at a high-volume tertiary care obstetric unit: a before-and-after study. Reg Anesth Pain Med. 2021 Feb;46(2):157-163. doi: 10.1136/rapm-2020-101873. Epub 2020 Nov 6. PMID: 33159006. | Wrong intervention |
| Emma Sprawson. Pain in labour and the intrapartum use of intramuscular opioids—how effective are they? British Journal of Midwifery 2017 25:7, 418-424 | Editorials, letters, or literature review |
| Epsztein Kanczuk M, Barrett NM, Arzola C, Downey K, Ye XY, Carvalho JC. Programmed Intermittent Epidural Bolus for Labor Analgesia During First Stage of Labor: A Biased-Coin Up-and-Down Sequential Allocation Trial to Determine the Optimum Interval Time Between Boluses of a Fixed Volume of 10 mL of Bupivacaine 0.0625% With Fentanyl 2 μg/mL. Anesth Analg. 2017 Feb;124(2):537-541. doi: 10.1213/ANE.0000000000001655. PMID: 27755057. | Wrong intervention |
| Fishburne JI. Systemic analgesia during labor. Clin Perinatol. 1982 Feb;9(1):29-53. PMID: 7039934. | Editorials, letters, or literature review |
| Fiszer E, Aptekman B, Baar Y, Weiniger CF. The effect of high-dose versus low-dose epidural fentanyl on gastric emptying in nonfasted parturients: A double-blinded randomised controlled trial. Eur J Anaesthesiol. 2022 Jan 1;39(1):50-57. doi: 10.1097/EJA.0000000000001514. PMID: 33852498. | Wrong intervention |
| Ginosar Y, Birnbach DJ, Shirov TT, Arheart K, Caraco Y, Davidson EM. Duration of analgesia and pruritus following intrathecal fentanyl for labour analgesia: no significant effect of A118G μ-opioid receptor polymorphism, but a marked effect of ethnically distinct hospital populations. Br J Anaesth. 2013 Sep 1;111(3):433–44. | Genetic study |
| Halpern SH, Abdallah FW. Effect of labor analgesia on labor outcome. Curr Opin Anaesthesiol. 2010 Jun;23(3):317-22. doi: 10.1097/ACO.0b013e3283385492. PMID: 20224384. | Wrong intervention |
| Haidl F, Rosseland LA, Spigset O, Dahl V. Effects of Adrenaline on maternal and fetal fentanyl absorption in epidural analgesia: A randomized trial. Acta Anaesthesiol Scand. 2018 Oct;62(9):1267-1273. doi: 10.1111/aas.13175. Epub 2018 Jun 25. PMID: 29943508; PMCID: PMC6174966. | Wrong intervention |
| Herrera-Gómez A, García-Martínez O, Ramos-Torrecillas J, De Luna-Bertos E, Ruiz C, Ocaña-Peinado FM. Retrospective study of the association between epidural analgesia during labour and complications for the newborn. Midwifery. 2015 Jun;31(6):613-6. doi: 10.1016/j.midw.2015.02.013. Epub 2015 Mar 11. PMID: 25819707. | Wrong intervention |
| Hurley RJ, Johnson MD. Spinal opioids in the management of obstetric pain. J Pain Symptom Manage. 1990 Jun;5(3):146-53. doi: 10.1016/0885-3924(90)90003-3. PMID: 2366009. | Editorials, letters, or literature review |
| Kavuri S, Janardhan Y, Fernando FE, Shevde K, Eddi D (1989) A comparative study of epidural alfentanil and fentanyl for labor pain relief. Anesthesiology 71, No3A | Wrong intervention |
| Kim DH, Kim TJ, Park NH. Comparison of Butorphanol and Fentanyl Administered with Bupivacaine for Patient Controlled Epidural Analgesia after Cesarean Section. Korean J Anesthesiol. 1998;34(1):126-131. | Wrong intervention |
| Kokki M., Heikkinen AT, Raatikainen K, Ranta VP, Hautajärvi H, Kokki H. Pharmacokinetics of intranasal fentanyl in parturient, BJA: British Journal of Anaesthesia, Volume 115, Issue 4, October 2015, Pages 635–636, <https://doi.org/10.1093/bja/aev313> | Pharmacokinetic study |
| Kuczkowski KM. Severe persistent fetal bradycardia following subarachnoid administration of fentanyl and bupivacaine for induction of a combined spinal-epidural analgesia for labor pain. J Clin Anesth. 2004 Feb;16(1):78-9. doi: 10.1016/j.jclinane.2003.12.004. PMID: 14984867. | Wrong intervention |
| Kumar, M., Paes, B. Epidural Opioid Analgesia and Neonatal Respiratory Depression. J Perinatol 23, 425–427 (2003). <https://doi.org/10.1038/sj.jp.7210905> | Case report |
| Kumar M, Chandra S, Ijaz Z, Senthilselvan A. Epidural analgesia in labour and neonatal respiratory distress: a case-control study. Arch Dis Child Fetal Neonatal Ed. 2014 Mar;99(2):F116-9. doi: 10.1136/archdischild-2013-304933. Epub 2013 Oct 29. PMID: 24170528. | Wrong intervention |
| Kutlesić M, Kutlesić R. [Epidural analgesia in labor: specific characteristics, dilemmas and controversies]. Med Pregl. 2012 Sep-Oct;65(9-10):441-7. Serbian. doi: 10.2298/mpns1210441k. PMID: 23214341. | Wrong intervention |
| Landau R, Liu SK, Blouin JL, Carvalho B. The effect of OPRM1 and COMT genotypes on the analgesic response to intravenous fentanyl labor analgesia. Anesth Analg. 2013 Feb;116(2):386-91. doi: 10.1213/ANE.0b013e318273f2c7. Epub 2013 Jan 9. PMID: 23302985. | Genetic study |
| Lange EMS, Wong CA, Fitzgerald PC, Davila WF, Rao S, McCarthy RJ, Toledo P. Effect of Epidural Infusion Bolus Delivery Rate on the Duration of Labor Analgesia: A Randomized Clinical Trial. Anesthesiology. 2018 Apr;128(4):745-753. doi: 10.1097/ALN.0000000000002089. PMID: 29351097. | Wrong intervention |
| Lee A, Landau R, Lavin T, Goodman S, Menon P, Smiley R. Comparative efficacy of epidural clonidine versus epidural fentanyl for treating breakthrough pain during labor: a randomized double-blind clinical trial. Int J Obstet Anesth. 2020 May;42:26-33. doi: 10.1016/j.ijoa.2019.11.003. Epub 2019 Nov 11. PMID: 31787454. | Wrong intervention |
| Lee A, Landau R, Lavin T, Goodman S, Menon P, Smiley R. Comparative efficacy of epidural clonidine versus epidural fentanyl for treating breakthrough pain during labor: a randomized double-blind clinical trial. Int J Obstet Anesth. 2020 May;42:26-33. doi: 10.1016/j.ijoa.2019.11.003. Epub 2019 Nov 11. PMID: 31787454. | Wrong intervention |
| Lee SH, Barcohana Y. Obstetric anesthesia and analgesia: options for pain relief during childbirth. Med Health R I. 2001 Oct;84(10):329-31. PMID: 11693051. | Editorials, letters, or literature review |
| Li RX, Ke SG, Xu XJ, Zhu TH (2013) Clinical analysis of effect of combined spinal-epidural in labor analgesia. Anhui Med 2(7):949–951. https://doi.org/10.3969/j.issn.1000-0399.2011.07.029 | Wrong intervention |
| Li CF, Deng JL, Shen LY (2013) Application of adding sufentanil to low concentration ropivacaine for combined spinal-epidural analgesia during labor. Youjiang Med 41(4):599–551. https://doi.org/10.3969/j.issn.1003-1383.2013.04.030 | Wrong intervention |
| Lilker S, Rofaeel A, Balki M, Carvalho JC. Comparison of fentanyl and sufentanil as adjuncts to bupivacaine for labor epidural analgesia. J Clin Anesth. 2009 Mar;21(2):108-12. doi: 10.1016/j.jclinane.2008.06.027. PMID: 19329014. | Wrong intervention |
| Lindow SW, Dhillon AR, Husaini SW, Russell IF. A randomised double-blind comparison of epidural fentanyl versus fentanyl and bupivicaine for pain relief in the second stage of labour. BJOG. 2004 Oct;111(10):1075-80. doi: 10.1111/j.1471-0528.2004.00267.x. PMID: 15383109. | Fentanyl was used during second stage of labour |
| Lopard E. L'analgésie péridurale obstétricale contrôlée par la patiente: en pratique [Patient-controlled epidural analgesia for labour: in practice]. Ann Fr Anesth Reanim. 2006 Jun;25(6):593-8. French. doi: 10.1016/j.annfar.2006.02.012. Epub 2006 Apr 19. PMID: 16626925. | Editorials, letters, or literature review |
| Lu YM, Lin GY, Zhang Y (2009) Effects of sufentanil and ropivacaine on labor analgesia combined with spinal-epidural analgesia. Chin Med 4(9):703–705. https://doi.org/10.3760/cma.j.issn.1673-4777.2009.09.029 | Wrong intervention |
| Macendo Amaral, Hudson Rodrigo, Sarmento Filho, Edson Diniz, Matos Silva, Diana, de Andrade Barbosa, Thiago Luis, & Xavier Gomes, Ludmila Mourão. (2015). Repercussões maternas e fetais da analgesia obstétrica: uma revisão integrativa. Avances en Enfermería, 33(2), 282-294. https://doi.org/10.15446/av.enferm.v33n2.52176 | Editorials, letters, or literature review |
| Manjunath C, Jacob R. A Comparison of Analgesia and Foetal Outcome in Term Parturients With and Without Low Dose Combined Spinal Epidural Labour Analgesia. J Evol Med Dent Sci. 2015;4(90):15589–92. | Wrong intervention |
| Mauri PA, Contini NN, Giliberti S, Barretta F, Consonni D, Negri M, Di Benedetto I. Intrapartum epidural analgesia and onset of lactation: a prospective study in an Italian birth centre. Matern Child Health J. 2015 Mar;19(3):511-8. doi: 10.1007/s10995-014-1532-x. PMID: 24894732. | Wrong intervention |
| Minty RG, Kelly L, Minty A, Hammett DC. Single-dose intrathecal analgesia to control labour pain: is it a useful alternative to epidural analgesia? Can Fam Physician. 2007 Mar;53(3):437-42. PMID: 17872679; PMCID: PMC1949078. | Editorials, letters, or literature review |
| Nikfar S, Rad RP, Soltani S, Taheri YS, Kamali A. Comparison of low-dose ketamine intravenous infusion with spinal anesthesia in pain control of mothers being a candidate for painless labor. 2021;36(1):50–5. | Wrong intervention |
| Norris MC. Intrathecal opioids and fetal bradycardia: is there a link? Int J Obstet Anesth. 2000 Oct 1;9(4):264–9. | Editorials, letters, or literature review |
| Ojo OA, Mehdiratta JE, Gamez BH, Hunting J, Habib AS. Comparison of Programmed Intermittent Epidural Boluses With Continuous Epidural Infusion for the Maintenance of Labor Analgesia: A Randomized, Controlled, Double-Blind Study. Anesth Analg. 2020 Feb;130(2):426-435. doi: 10.1213/ANE.0000000000004104. PMID: 30882524. | Wrong intervention |
| Okutomi T, Saito M, Mochizuki J, Amano K, Hoka S. A double-blind randomized controlled trial of patient-controlled epidural analgesia with or without a background infusion following initial spinal analgesia for labor pain. Int J Obstet Anesth. 2009 Jan;18(1):28-32. doi: 10.1016/j.ijoa.2008.06.006. Epub 2008 Nov 20. PMID: 19022653. | Wrong intervention |
| Paech MJ. Patient controlled epidural analgesia during labour: choice of solution. Int J Obstet Anesth. 1993;2(2):65-71. doi: 10.1016/0959-289x(93)90081-r. PMID: 15636853. | Wrong intervention |
| Paulina Cortes, Jaime Molina, Dagoberto Ojeda, Patricia Cisternas. Programmed intermittent bolus epidural analgesia for obstetrics: a comparison of two regimens. Anesthesia & Analgeaia September 2021. Vol 133. Issue 3. Supplement 2 | Wrong intervention |
| Prommer E, Thompson L. Intranasal fentanyl for pain control: current status with a focus on patient considerations. Patient Prefer Adherence. 2011;5:157-164 <https://doi.org/10.2147/PPA.S7665> | Editorials, letters, or literature review |
| Radzyminski S. The effect of ultra low dose epidural analgesia on newborn breastfeeding behaviors. J Obstet Gynecol Neonatal Nurs. 2003 May-Jun;32(3):322-31. doi: 10.1177/0884217503253440. PMID: 12774874. | Wrong intervention |
| Reem Hatamleh, Reem Ali, Amira Ishaq Elian. Epidural analgesia and its effects on maternal and neonatal outcomes: a retrospective comparable study in Northern Jordan. Evidence Based Midwifery; London Vol. 17, Iss. 4, (Dec 2019): 135-142. | Wrong intervention |
| Reynolds F. The effects of maternal labour analgesia on the fetus. Best Pract Res Clin Obstet Gynaecol. 2010 Jun;24(3):289-302. doi: 10.1016/j.bpobgyn.2009.11.003. Epub 2009 Dec 11. PMID: 20005180. | Editorials, letters, or literature review |
| Riordan J, Gross A, Angeron J, Krumwiede B, Melin J. The effect of labor pain relief medication on neonatal suckling and breastfeeding duration. J Hum Lact. 2000 Feb;16(1):7-12. doi: 10.1177/089033440001600103. PMID: 11138228. | Wrong intervention |
| Rodríguez-Campoó MB, Curto A, González M, Aldecoa C. Patient intermittent epidural boluses (PIEB) plus very low continuous epidural infusion (CEI) versus patient-controlled epidural analgesia (PCEA) plus continuous epidural infusion (CEI) in primiparous labour: a randomized trial. J Clin Monit Comput. 2019 Oct;33(5):879-885. doi: 10.1007/s10877-018-0229-x. Epub 2018 Nov 30. PMID: 30506299. | Wrong intervention |
| Russell R, Reynolds F. Epidural infusions for nulliparous women in labour. A randomised double-blind comparison of fentanyl/bupivacaine and sufentanil/bupivacaine. Anaesthesia. 1993 Oct;48(10):856-61. doi: 10.1111/j.1365-2044.1993.tb07413.x. PMID: 8238826. | Wrong intervention |
| Sakr A, Cohen S, Chiricolo A, Ramos D, Rah K, Doucette A. Does increasing the concentration of epidural-PCA fentanyl for labor improve analgesia without effect to neonate? Anesth Analg. 2014;118(5 SUPPL. 1):S187. | Wrong intervention |
| Scherer R, Holzgreve W. Influence of epidural analgesia on fetal and neonatal well-being. Eur J Obstet Gynecol Reprod Biol. 1995 May;59 Suppl:S17-29. doi: 10.1016/0028-2243(95)93909-8. PMID: 7556818. | Editorials, letters, or literature review |
| Sezer OA, Gunaydin B, Sezer OA, Gunaydin B. Efficacy of patient-controlled epidural analgesia after initiation with epidural or combined spinal-epidural analgesia. Int J Obstet Anesth. 2007 Jul;16(3):226–30. | Wrong intervention |
| Sharma SK, McIntire DD, Wiley J, Leveno KJ. Labor analgesia and cesarean delivery: an individual patient meta-analysis of nulliparous women. Anesthesiology. 2004 Jan;100(1):142-8; discussion 6A. doi: 10.1097/00000542-200401000-00023. PMID: 14695735. | Wrong intervention |
| Shum S, Shen DD, Isoherranen N. Predicting Maternal-Fetal Disposition of Fentanyl Following Intravenous and Epidural Administration Using Physiologically Based Pharmacokinetic Modeling. Drug Metab Dispos. 2021 Nov;49(11):1003-1015. doi: 10.1124/dmd.121.000612. Epub 2021 Aug 18. PMID: 34407992. | Pharmacokinetic study |
| Sia AT, Leo S, Ocampo CE. A randomised comparison of variable-frequency automated mandatory boluses with a basal infusion for patient-controlled epidural analgesia during labour and delivery. Anaesthesia. 2013 Mar;68(3):267-75. doi: 10.1111/anae.12093. Epub 2012 Dec 20. PMID: 23278328. | Wrong intervention |
| Skupski DW, Abramovitz S, Samuels J, Pressimone V, Kjaer K. Adverse effects of combined spinal-epidural versus traditional epidural analgesia during labor. Int J Gynaecol Obstet. 2009 Sep;106(3):242-5. doi: 10.1016/j.ijgo.2009.04.019. Epub 2009 May 28. PMID: 19481203. | Wrong intervention |
| Sng BL, Sia ATH. Maintenance of epidural labour analgesia: The old, the new and the future. Best Pract Res Clin Anaesthesiol. 2017 Mar;31(1):15-22. doi: 10.1016/j.bpa.2017.01.002. Epub 2017 Jan 12. PMID: 28625301. | Editorials, letters, or literature review |
| Sng BL, Zhang Q, Leong WL, Ocampo C, Assam PN, Sia AT. Incidence and characteristics of breakthrough pain in parturients using computer-integrated patient-controlled epidural analgesia. J Clin Anesth. 2015 Jun;27(4):277-84. doi: 10.1016/j.jclinane.2015.01.003. Epub 2015 Feb 14. PMID: 25690278. | Wrong intervention |
| Song Z, Du B, Wang K, Shi X. Effects of OPRM1 A118G polymorphism on epidural analgesia with fentanyl during labor: a meta-analysis. Genet Test Mol Biomarkers. 2013 Oct;17(10):743-9. doi: 10.1089/gtmb.2013.0282. Epub 2013 Aug 2. PMID: 23909491. | Genetic study |
| Soo-Jung Han, Jeung-Im Kim, Myo-Jin Kim. Comparison of Obstetric Pain, Anxiety, and Cervical Dilatation between Epidural Analgesia and No Analgesia group during Labor Stage I. 2012 Korean Journal of Women Health Nursing 2012; 18(2): 126-134. DOI: <https://doi.org/10.4069/kjwhn.2012.18.2.126> | Wrong intervention |
| Staikou C, Kalampokas T, Kalampokas E, Vassiloglou S, Paraskeva A. Epidural fentanyl does not affect cervical dilation and progress of first stage of vaginal delivery: a randomized, double-blind study. Curr Med Res Opin. 2017 Aug;33(8):1491-1496. doi: 10.1080/03007995.2017.1321536. Epub 2017 May 24. PMID: 28425307. | Wrong intervention |
| Sullivan JT, Grobman WA, Bauchat JR, Scavone BM, Grouper S, McCarthy RJ, Wong CA. A randomized controlled trial of the effect of combined spinal-epidural analgesia on the success of external cephalic version for breech presentation. Int J Obstet Anesth. 2009 Oct;18(4):328-34. doi: 10.1016/j.ijoa.2009.02.006. Epub 2009 Aug 13. PMID: 19682886. | Fentanyl was used for external cephalic version of breech presentation |
| Sweed N, Sabry N, Azab T, Nour S. Regional versus IV analgesics in labor. Minerva Med. 2011 Oct;102(5):353-61. PMID: 22193345. | Wrong intervention |
| Tang ZJ, Huang N, Shu JJ. (2017) Comparison of the effects of ropivacaine combined with sufentanil or fentanyl under CSEA combined with PCEA for labor analgesia. China practical medicine 12(19):124-6. https://doi.org/10.14163/j.cnki.11-5547/r.2017.19.  066 | Wrong intervention |
| The most important goal in managing labour pain is patient satisfaction. (2010). Drugs and Therapy Perspectives, 26(3), 15–17. <https://doi.org/10.2165/11204120-000000000-00000> | Editorials, letters, or literature review |
| Use of epidurals in labour. (2019). World of Irish Nursing & Midwifery, 27(9), 42. | Editorials, letters, or literature review |
| Vilda Baliuliene, Andrius Macas, Kestutis Rimaitis. Safety and efficacy of different local anaesthetics and their concentrations, used for labour pain relief with PCEA for healthy primiparas: a randomized double blind controlled trial. 2017 The Acta Anaesthesiologica Scandinavica Foundataion 61.962-1062 | Wrong intervention |
| Villecco D. (2007). Evidence based anesthesia: fever of unknown origin in the parturient and neuraxial anesthesia. AANA Journal., 75(5), 386–387. | Editorials, letters, or literature review |
| Viscomi CM, Rathmell JP, Pace NL. Duration of intrathecal labor analgesia: early versus advanced labor. Anesth Analg. 1997 May;84(5):1108-12. doi: 10.1097/00000539-199705000-00028. PMID: 9141940. | Wrong intervention |
| Wassen MM, Smits LJ, Scheepers HC, Marcus MA, Van Neer J, Nijhuis JG, Roumen FJ. Routine labour epidural analgesia versus labour analgesia on request: a randomised non-inferiority trial. BJOG. 2015 Feb;122(3):344-50. doi: 10.1111/1471-0528.12854. Epub 2014 May 22. PMID: 24849943. | Wrong intervention |
| Weissman, A., Torkhov, O., Weissman, A. I., & Drugan, A. (2009). The effects of meperidine and epidural analgesia in labor on maternal heart rate variability. International Journal of Obstetric Anesthesia, 18(2), 118–124. <https://doi.org/10.1016/j.ijoa.2008.09.006> | Wrong intervention |
| Wilson DJ, Douglas MJ. Neuraxial opioids in labour. Baillieres Clin Obstet Gynaecol. 1998 Sep;12(3):363-76. doi: 10.1016/s0950-3552(98)80072-2. PMID: 10023426. | Editorials, letters, or literature review |
| Wong CA, Peaceman AM. Effect of early epidural analgesia on labor: Cutting through the confusion. Contemp Ob Gyn. 2006;51(7):64–70. | Wrong intervention |
| Wong CA, Scavone BM, Peaceman AM, McCarthy RJ, Sullivan JT, Diaz NT, et al. The Risk of Cesarean Delivery with Neuraxial Analgesia Given Early versus Late in Labor. N Engl J Med. 2005 Feb 17;352(7):655–65. | Wrong intervention |
| Wong CA. The influence of analgesia on labor--is it related to primary cesarean rates? Semin Perinatol. 2012 Oct;36(5):353-6. doi: 10.1053/j.semperi.2012.04.019. PMID: 23009968. | Editorials, letters, or literature review |
| Wong CA, McCarthy RJ, Blouin J, Landau R. Observational study of the effect of mu-opioid receptor genetic polymorphism on intrathecal opioid labor analgesia and post-cesarean delivery analgesia. Int J Obstet Anesth. 2010 Jul;19(3):246-53. doi: 10.1016/j.ijoa.2009.09.005. Epub 2010 Feb 19. PMID: 20171873. | Genetic study |
| Wu C, Shi B, Jiang H. Clinical observation of regular intermittent epidural injection combined with different puncture points in suppressing breakthrough pain in labour analgesia. J Obstet Gynaecol. 2019 Apr;39(3):297-301. doi: 10.1080/01443615.2018.1469604. Epub 2018 Nov 8. PMID: 30406725. | Wrong intervention |
| Yan XB, Li SQ (2011) Effects of sufentanil to ropivacaine for combined spinal-epidural analgesia during labor. Matern Child Healthc Chin 26(28):4466–4468 ISSN: 1001-4411 (2011)28-4466-03 | Wrong intervention |
| Yong In Kang, Myoung Hee Kim, Su Yeon Kim, Eun Chi Bang, Hyun Sook Lee, Kyoung Sook Cho, Su Yeon Kim. Effect of Continuous Epidural Analgesia on the Progress of Labor. Korean J Anesthesiol. 2000;39(2):183-188. DOI: https://doi.org/10.4097/kjae.2000.39.2.183 | Wrong intervention |
| Younsuk Lee, Youngkeun Chae, Youngjoon Oh, Haekyoung Kim, Choonkun Chung. Patient-Controlled Epidural Analgesia during Labor and Delivery. Korean J Anesthesiol. 1997;32(2):274-280.  DOI: https://doi.org/10.4097/kjae.1997.32.2.274 | Wrong intervention |
| Zaphiratos V, George RB, Macaulay B, Bolleddula P, McKeen DM. Epidural Volume Extension During Combined Spinal-Epidural Labor Analgesia Does Not Increase Sensory Block. Anesth Analg. 2016 Sep;123(3):684-9. doi: 10.1213/ANE.0000000000001281. PMID: 27088994. | Wrong intervention |
| Zgheib NK, Aouad MT, Taha SK, Nassar AH, Masri RF, Khoury MY, Makki MH, Siddik-Sayyid SM. μ-opioid receptor genetic polymorphisms and duration of epidural fentanyl analgesia during early labor. Minerva Anestesiol. 2018 Aug;84(8):946-954. doi: 10.23736/S0375-9393.18.12697-6. Epub 2018 May 14. PMID: 29756748. | Genetic study |
| Zheng Lei, Wu Lijun. Comparison of adding sufentanil or fentanyl to ropivacaine for combined spinal-epidural analgesia during labor [J]. Anhui Medicine, 2016,20(3):576-577,578. DOI: 10.3969/j.issn.1009-6469.2016.03.052. | Wrong intervention |
| Zheng Jianqiu, Feng Jifeng, Peng Wei, et al. Clinical observation of sufentanil and fentanyl for combined spinal-epidural labor analgesia [J]. Guangxi Medicine, 2009,31(9):1267-1269. DOI: 10.3969/j.issn.0253-4304.2009.09.018. | Wrong intervention |
| Zuokumor, P., & Columb, M. (2003). Epidural anaesthesia for pain relief in labour. European Journal of Anaesthesiology, 20(8), 674-675. doi:10.1017/S0265021503211091 | Editorials, letters, or literature review |

**Supplementary table S5.** Characteristics of included studies

| **ID** | **Title** | **Author(s)** | **Year** | **Country** | **Study design** | **Sample size** | **Description of fentanyl** | **Reported outcome** |
| --- | --- | --- | --- | --- | --- | --- | --- | --- |
| 1 | Intrathecal bupivacaine reduces pruritus and prolongs duration of fentanyl analgesia during labor: A prospective, randomized controlled trial | Asokumar B, Michael Newman L, McCarthy RJ, Ivankovich AD, Tuman KJ. | 1998 | USA | RCT | 61 | Intrathecal 25 µg of fentanyl (0.5 mL) + 1.5 mL of preservative-free isotonic sodium chloride solution. No additional medications were administered until the patient requested further analgesia. | Pain score, maternal assessment (BP, HR, motor, sensory), delivery (induction), analgesia (duration, pruritus), satisfaction, neonatal assessment (FHR) |
| 2 | Double-blind comparison of intravenous butorephanol (Stadol) and fentanyl (Sublimaze) for analgesia during labor | Atkinson BD, Truitt LJ, Rayburn WF, Turnbull GL, Dix Christensen H, Wlodaver A. | 1994 | USA | RCT | 50 | Standard intravenous doses were prescribed for fentanyl, 50 to 100 µg every 1 to 2 hours. | Pain score, maternal assessment (RR), delivery (mode, duration, contraction, vomiting, sedation), neonatal assessment (FHR, apgar, cord blood gases, naloxone requirement, Neurologic & Adaptive Capacity Score, resuscitation) |
| 3 | Minimum analgesic doses of fentanyl and sufentanil for epidural analgesia in the first stage of labor | Capogna G, Camorcia M, Columb MO. | 2003 | Italy | RCT | 62 | Each woman received a 10-mL volume of epidural drug that comprised fentanyl (Fentanest; Pharmacia & Upjohn) diluted with 0.9% wt/vol saline to achieve the desired dose. Starting dose was arbitrarily chosen to be 125 µg for fentanyl. | Pain score, maternal assessment (BP, HR, SPO2), SE (nausea, vomiting, pruritus, sedation), neonatal assessment (FHR) |
| 4 | Effect of intrathecal fentanyl dose on the duration of labor analgesia | Celeski DC, Heindel L, Haas J, Vacchiano CA. | 1999 | USA | RCT | 56 | 25, 37.5, 50 µg of intrathecal fentanyl was randomly administered by mixing with preservative free normal saline 0.9% to achieve a consistent volume of 2mL. | Pain score, maternal assessment (BP, RR, induction, contraction), analgesia (duration, additional analgesia), SE (nausea, pruritus), neonatal assessment (FHR) |
| 5 | Comparison of epidural fentanyl versus epidural sufentanil for analgesia in ambulatory patients in early labor | Connelly NR, Dunn S, Connelly NR, Parker RK, Vallurupalli V, Bhopatkar S, et al. | 2000 | USA | RCT | 45 | Each patient received 1000 mL of lactated Ringers solution IV. The patient was given epidural fentanyl 100 µg with normal saline to a total volume of 10 mL. | Pain score, maternal assessment (BP, HR, RR, motor), delivery (mode, duration), analgesia (duration, additional analgesia), SE (nausea, vomiting, pruritus, sedation, headache), neonatal assessment (FHR, apgar) |
| 6 | The influence of a bupivacaine and fentanyl epidural infusion after epidural fentanyl in patients allowed to ambulate in early labor | Connelly NR, Parker RK, Lucas T, El-Mansouri M, Komanduri V, Nayak P, et al. | 2001 | USA | RCT | 50 | The patient was given epidural fentanyl 100 µg diluted in normal saline to a total volume of 10 mL. | Pain score, maternal assessment (BP, HR, RR, motor), delivery (mode, duration), analgesia (duration, additional analgesia), SE (nausea, vomiting, pruritus, sedation, headache), neonatal assessment (FHR, apgar) |
| 7 | Labour analgesia with intrathecal fentanyl decreases maternal stress | Cascio M, Pygon B, Bernett C, Ramanathan S. | 1997 | USA | RCT | 24 | The patient was given 500 ml Ringers IV solution prior to initiation of analgesia. Then, patients received 25 μg intrathecal fentanyl. | Pain score, maternal assessment (BP, HR), SE (pruritus), maternal stress |
| 8 | Obstetric analgesia: a comparison of patient-controlled meperidine, remifentanil, and fentanyl in labour | Douma MR, Verwey RA, Kam-Endtz CE, van der Linden PD, Stienstra R. | 2010 | Netherlands | RCT | 180 | Women in the fentanyl group (Group F) received a 50 µg loading dose and boluses of 20 µg with a lockout of 5 min and a maximum dose limit of 240 µg h^-1^. | Pain score, maternal assessment (BP, HR, RR, SPO2), delivery (mode, duration, induction), analgesia (duration, additional analgesia), SE (nausea, vomiting, pruritus), neonatal assessment (FHR, apgar, cord blood gases, naloxone requirement, Neurologic & Adaptive Capacity Score, resuscitation) |
| 9 | Taking the alternative route: Women’s experience of intranasal fentanyl, subcutaneous fentanyl or intramuscular pethidine for labour analgesia | Fleet JA, Jones M, Belan I. | 2017 | Australia | Qualitative study | 116 | Healthy women birthing at term, who received intranasal fentanyl, subcutaneous fentanyl and/or intramuscular  Pethidine for labour analgesia, were contacted at 6 weeks postpartum to complete a phone questionnaire. | Breastfeeding problem, satisfaction |
| 10 | A comparison of fentanyl with pethidine for pain relief during childbirth: a randomised controlled trial | Fleet J, Belan I, Jones MJ, Ullah S, Cyna AM. | 2015 | Australia | RCT | 144 | - Participants received a 200 microgram bolus dose of fentanyl administered subcutaneously. After 1 hour, additional 50 microgram doses could be administered every 15 minutes, as requested, up to a maximum of 650 micrograms. - Participants self-administered a 54 microgram (0.18 ml) fentanyl dose sprayed into the nose using a patientcontrolled intranasal analgesia (PCINA) device (Go Medical Industries, Perth, Western, Australia). This device had a 4 minute filling time that acted as a lockout between doses. The maximum hourly dose was 600 micrograms, with a maximum total dose of 1200 micrograms. | Pain score, maternal assessment (BP, HR, RR, SPO2), delivery (mode, duration, postpartum hospital stay), SE (vomiting, sedation), breastfeeding problem, satisfaction, neonatal assessment (apgar, cord blood gases, birthweight, naloxone requirement, nursery admission, time to establish breathing) |
| 11 | Subcutaneous administration of fentanyl in childbirth: an observational study on the clinical effectiveness of fentanyl for mother and neonate | Fleet J. | 2011 | Australia | Retrospective cohort | 418 | Medical records of 418  labouring women birthing within one rural hospital during  January 2000—December 2007, were examined to explore the clinical effectiveness of subcutaneously administered fentanyl. Then, A pilot study (n = 10) was conducted between July 2008 and October 2008 to assess the efficacy of subcutaneously administered fentanyl for pain relief during childbirth. | Pain score, breastfeeding problem, neonatal assessment (apgar) |
| 12 | The influence of intrapartum opioid use on breastfeeding experience at 6 weeks post partum: A secondary analysis | Fleet JA, Jones M, Belan I. | 2017 | Australia | Prospective cohort | 109 | At six weeks postpartum women were contacted by telephone to participate in a  questionnaire that examined factors that are recognised to impact on breastfeeding outcomes | Delivery (mode, induction, postpartum hospital stay), breastfeeding problem, neonatal assessment (apgar, cord blood gases, birthweight, nursery admission, time to establish breathing) |
| 13 | Fentanyl concentration in maternal and umbilical cord plasma following intranasal or subcutaneous administration in labour | Fleet J-A, Belan I, Gordon AL, Cyna AM. | 2020 | Australia | Prospective cohort | 30 | Maternal and cord  blood samples were collected within 30 min of birth to determine the fentanyl plasma concentration and to assess relative neonatal exposure. Neonatal outcomes were assessed by Apgar scores, need for resuscitation and nursery admission. | Delivery (mode, duration, induction), analgesia (fentanyl concentration), neonatal assessment (apgar, naloxone requirement, nursery admission, time to establish breathing) |
| 14 | Subcutaneous fentanyl for labour analgesia: a retrospective case note review | Fleet J, Sok C, Randall ER, Cyna AM. | 2021 | Australia | Retrospective descriptive study | 102 | Case notes for the first 100 women administered subcutaneous fentanyl during labour were reviewed. | Delivery (mode, duration, induction, postpartum hospital stay), analgesia (additional analgesia), neonatal assessment (apgar, cord blood gases, birthweight, resuscitation, nursery admission) |
| 15 | Comparison of three different doses of intrathecal fentanyl and sufentanil for labor analgesia | Gaiser RR, Cheek TG, Gutsche BB | 1998 | USA | RCT | 55 | Patients received intrathecal study solutions: fentanyl 25, 37.5, or 50 µg. All study  solutions were diluted with normal saline to achieve a  volume of 1.5 ml. | Pain score, maternal assessment (BP, HR, SPO2, delivery (contraction), analgesia (additional analgesia), SE (pruritus), neonatal assessment (FHR, apgar, cord blood gases, Neurologic & Adaptive Capacity Score) |
| 16 | Epinephrine is not a useful addition to intrathecal fentanyl or fentanyl-bupivacaine for labor analgesia | Goodman SR, Kim-Lo SH, Ciliberto CF, Ridley DM, Smiley RM. | 2002 | USA | RCT | 76 | Intrathecal solutions containing fentanyl 35 µg (0.7ml) with 1.1 ml saline was given. | Pain score, maternal assessment (BP, HR, motor, sensory), delivery (mode), analgesia (duration), SE (nausea, vomiting, pruritus), neonatal assessment (FHR, birthweight) |
| 17 | A multicenter randomized controlled trial comparing patient-controlled epidural with intravenous analgesia for pain relief in labor | Halpern SH, Blanchard JW, Halpern SH, Muir H, Breen TW, Campbell DC, et al. | 2004 | Canada | RCT | 242 | Given an initial IV dose of 100 µg of fentanyl incrementally over 1–5 min. If the pain was not adequately relieved, an additional 50 µg was given and repeated every 5 min until the patient reported adequate pain relief. | Pain score, maternal assessment (RR, motor, fever), delivery (mode, duration, induction), SE (vomiting, sedation), satisfaction, neonatal assessment (apgar, cord blood gases, birthweight, naloxone requirement, resuscitation, fever) |
| 18 | Analgesia, pruritus, and ventilation exhibit a dose-response relationship in parturients receiving intrathecal fentanyl during labor | Herman NL, Choi KC, Affleck PJ, Calicott R, Brackin R, Singhal A, et al. | 1999 | USA | RCT | 90 | Received one of six doses of intrathecal fentanyl: 5, 7.5, 10, 15, 20, or 25 µg. | Pain score, maternal assessment (BP, RR, motor, sensory, SPO2), delivery (contraction), SE (nausea, vomiting, pruritus), neonatal assessment (FHR, birthweight) |
| 19 | Comparison among intrathecal fentanyl, meperidine, and sufentanil for labor analgesia. | Honet JE, Arkoosh VA, Norris MC, Huffnagle HJ, Silverman NS, Leighton BL. | 1992 | USA | RCT | 60 | A 1 ml solution containing 10 μg fentanyl in preservative free normal saline was injected into the intrathecal splace. | Pain score, maternal assessment (BP, motor, sensory), delivery (mode, duration, contraction), analgesia (duration), SE (nausea, headache), neonatal assessment (FHR, apgar, cord blood gases) |
| 20 | Retrospective evaluation of intravenous fentanyl patient-controlled analgesia during labor | Hosokawa Y, Morisaki H, Nakatsuka I, Hashiguchi S, Miyakoshi K, Tanaka M, et al. | 2012 | Japan | Retrospective cohort | 840 | On the parturients’ first request for analgesia during labor, 0.05 mg fentanyl was injected as a loading dose, followed by the application of an iv-PCA pump (i-Fusor; JMS, Tokyo, Japan) that did not deliver constant flow, but only bolus doses of 0.02 mg, with a lock-out interval of 5 min. The  maximum dose of fentanyl per hour could be increased up  to a maximum of 0.24 mg, in total. | Maternal assessment (HR, RR, SPO2), delivery (mode, duration, induction), analgesia (duration), SE (nausea, vomiting, sedation), neonatal assessment (FHR, apgar, cord blood gases, birthweight, naloxone requirement, resuscitation) |
| 21 | The effect of intravenous fentanyl on pain and duration of the active phase of first stage labor. | Jahani Shoorab N, Ebrahimzadeh Zagami S, Mirzakhani K, Mazlom SR. | 2013 | Iran | RCT | 70 | IV 50 micrograms fentanyl was prescribed in two doses with an interval of 1 hour after being diluted in 4 cc normal  saline (total volume 5 cc - 25 µg /5 ml during 10 minutes infusion and repeated second dose an hour later 25 µg /5 ml) at zero and 60 minutes. | Pain score, maternal assessment (BP, HR, RR), delivery (duration, contraction), SE (nausea, vomiting, pruritus, sedation), neonatal assessment (FHR, apgar, resuscitation) |
| 22 | Patient-controlled intranasal fentanyl analgesia: a pilot study to assess practicality and tolerability during childbirth | Kerr D, Taylor D, Evans B, Kerr D TD, Evans B. | 2015 | Australia | Quasi-experimental study | 32 | Intranasal fentanyl 54 µg at 3-min intervals with no loading dose. | Pain score, maternal assessment (BP, HR, RR), delivery (mode, duration), analgesia (duration), SE (nausea, vomiting, sedation, headache, nasal irritation), neonatal assessment (FHR, apgar, birthweight, CPAP requirement) |
| 23 | Neonatal safety of maternal fentanyl during labour | Kokki M, Westeren-Punnonen S, Hautajärvi H, Heinonen S, Mazzei M, Määttä S, et al. | 2015 | Finland | RCT | 49 | Given intranasal fentanyl 100–250 µg (Instanyl® nasal spray 50 µg dose−1, Oy Leiras Takeda Pharmaceuticals Ab, Helsinki, Finland) when contraction pain was >5/10 (numerical rating scale (NRS) 0=no pain, 10=most pain). | Delivery (mode), analgesia (fentanyl concentration), neonatal assessment (FHR, apgar, cord blood gases, Neurologic & Adaptive Capacity Score) |
| 24 | Addition of bupivacaine 1.25 mg to fentanyl confers no advantage over fentanyl alone for intrathecal analgesia in early labour | Lim EHL, Sia ATH, Wong K Tan HM | 2002 | Singapore | RCT | 48 | 25 µg of fentanyl were diluted with preservative free normal  saline solution to a total volume of 2 mL and administered intrathecally over 30 sec. | Pain score, maternal assessment (BP, motor, sensory), analgesia (duration), SE (nausea, vomiting, pruritus, sedation, shivering), neonatal assessment (FHR) |
| 25 | Does fentanyl epidural analgesia affect breastfeeding: A prospective cohort study | Mahomed K, Wild K, Brown C, Green A. | 2019 | Australia | Prospective cohort | 304 | Women who had fentanyl epidural only were compared with women who had subcutaneous morphine only, and women who had neither subcutaneous morphine nor epidural. | Delivery (mode), SE (PPH), breastfeeding problem, neonatal assessment (birthweight, nursery admission) |
| 26 | Perinatal outcomes: Intravenous patient-controlled fentanyl versus no analgesia in labor | Miyakoshi K, Tanaka M, Morisaki H, Kim S-H, Hosokawa Y, Matsumoto T, et al. | 2013 | Japan | Retrospective cohort | 1301 | Loading dose, 50 μg iv fentanyl; bolus injection, 20 μg iv fentanyl; lock-out period, 5 min. The maximum dose of fentanyl per hour could be increased up to a maximum of 240 μg in total. | Pain score, maternal assessment (BP, HR, RR, SPO2), delivery (mode, duration, induction, contraction), SE (nausea, vomiting, sedation), neonatal assessment (FHR, apgar, cord blood gases, birthweight, naloxone requirement) |
| 27 | Comparison of intrathecal injection of fentanyl and sufentanil on the onset, duration, and quality of analgesia in labor: a randomized, double-blind clinical trial | Manouchehrian N RSMA,  Lakpur Z, Manouchehrian N, Rabiei S, Moradi A, Lakpur Z. | 2020 | Iran | RCT | 53 | Patients received 1.5 mL (75 µg) fentanyl (Feniject, Aburehan, Iran) intrathecal injection. | Pain score, maternal assessment (BP, HR, RR, SPO2), delivery (mode, duration), analgesia (duration), SE (nausea, vomiting, pruritus, sedation, shivering), neonatal assessment (FHR, apgar) |
| 28 | Remifentanil versus fentanyl for intravenous patient-controlled labour analgesia: An observational study | Marwah R, Hassan S, Carvalho JCA, Balki M. | 2012 | Canada | Retrospective cohort | 98 | Fentanyl PCA (diluted as 20 µg.mL^-1^) demand boluses 25-50 µg with a lockout interval of three to six minutes, a four-hour limit of 1-1.5 mg, and without any background infusion. | Pain score, maternal assessment (BP, HR, RR, motor, SPO2), delivery (mode), analgesia (duration), SE (nausea, vomiting, pruritus, sedation, neonatal assessment (apgar, cord blood gases, birthweight, resuscitation, congenital anomalies) |
| 29 | A comparison of patient-controlled analgesia fentanyl and alfentanil for labour analgesia | Morley-forster PK, Reid DW, Vandeberghe H. | 2000 | United Kingdom | RCT | 23 | Patients received 50 µg fentanyl iv by prepared solution 10 µg·ml–1 fentanyl. For all patients, the Bard II PCA pump was programmed to deliver a dose of 2 ml with a delay of five minutes and a basal rate of 2 ml·hr– 1. The maximal hourly rate was set at 26 ml. | Pain score, delivery (mode), analgesia (duration, fentanyl concentration), SE (nausea, vomiting, sedation), satisfaction, neonatal assessment (apgar, cord blood gases, naloxone requirement, Neurologic & Adaptive Capacity Score) |
| 30 | Neonatal effects of patient-controlled analgesia using fentanyl in labor | Morley-Forster PK, Weberpals J. | 1998 | Canada | Retrospective descriptive study | 31 | The hospital records of all women who had used PCA fentanyl in labor between 1992 and 1994 were identified and reviewed. | Maternal assessment (BP), delivery (mode, induction), analgesia (duration), neonatal assessment (apgar, cord blood gases, birthweight, naloxone requirement) |
| 31 | Clinical experience of epidural fentanyl for labor pain | Murakawa K, Abboud TK, Yanagi T, Sarkis F, Afrasiabi A, Sheikh-ol-Eslam A, et al. | 1987 | Japan | Quasi-experimental study | 5 | All parturients received epidural 50 µg of fentanyl in 10 ml of normal saline through a lumbar epidural catheter. | Pain score, maternal assessment (vital signs), SE (subjective maternal SE), neonatal assessment (apgar, cord blood gases, Neurologic & Adaptive Capacity Score) |
| 32 | A comparison of intrathecal fentanyl and sufentanil for labor analgesia. Anesthesiology | Nelson KE, Rauch T, Terebuh V, D’Angelo R. | 2002 | USA | RCT | 55 | In phase I, the ED 50 of intrathecal fentanyl was estimated by duplicating a previous study design that estimated an ED50 for intrathecal sufentanil. In phase II, women received 36 µg intrathecal fentanyl. | Pain score, maternal assessment (BP, motor, sensory, vital signs), delivery (mode), analgesia (duration), SE (nausea, pruritus, sedation), neonatal assessment (FHR) |
| 33 | Blood pressure, but not cerebrospinal fluid fentanyl concentration, predicts duration of labor analgesia from spinal fentanyl | Nelson KE, Houle TT, Eisenach JC. | 2010 | USA | Descriptive | 52 | Intrathecal fentanyl (50 µg) diluted to a total volume of 3 ml with sterile saline was injected over 10 s while observing the clock. | Pain score, maternal assessment (BP, HR), analgesia (duration, fentanyl concentration) |
| 34 | Labor Analgesia when Neuraxial Anesthesia is Relatively Contraindicated : Comparison of Patient-Controlled Fentanyl and Intermittent Nalbuphine Boluses | Nguyen T-A, Wang X-F, Wagner K, Izquierdo M, Bolden N. | 2018 | USA | Retrospective cohort | 104 | An initial bolus of 50-100 mcg of IV fentanyl with initial PCA settings allowing 50 mcg bolus doses every 10 minutes with an initial 1 hour maximum dose 250 mcg. | Pain score, delivery (duration, induction), SE (bag mask ventilation, intubation, naloxone, SPO2<90), neonatal assessment (naloxone requirement, resuscitation, CPAP requirement, epinephrine requirement) |
| 35 | Intravenous fentanyl PCA during labour | Nikkola EM, Ekblad UU, Kero PO, Alihanka JIM, Salonen MAO. | 1997 | Finland | RCT | 20 | A loading dose of 50 µg fentanyl was initially injected. Then, a patient-controlled pump (Graseby 3300) was set to deliver bolus doses of 20 µg fentanyl with a lock-out period of five minutes. The maximum dose in one hour was 240 µg. | Pain score, maternal assessment (HR, RR, SPO2), delivery (duration), analgesia (fentanyl concentration), SE (nausea, vomiting, sedation, shivering, neurological symptoms), satisfaction, neonatal assessment (FHR, apgar, cord blood gases, Neurologic & Adaptive Capacity Score, SPO2, body movement) |
| 36 | Neonatal Monitoring after Maternal Fentanyl Analgesia in Labor | Nikkola EM, Jahnukainen TJ, Ekblad UU, Kero PO, Salonen MAO. | 2001 | Finland | RCT | 12 | The mothers were initially given 50 μg IV fentanyl every 5 minutes until their pain was relieved or side effects appeared. Then, patient controlled pump was set to deliver boluses of 50 μg fentanyl with a lock out period of 5 minutes. | Pain score, maternal assessment (BP, HR, SPO2), analgesia (duration, fentanyl concentration), SE (subjective maternal SE), neonatal assessment (FHR, cord blood gases, birthweight, naloxone requirement, Neurologic & Adaptive Capacity Score, fever, SPO2, cyanosis) |
| 37 | The association between intrapartum opioid fentanyl and early breastfeeding: A prospective observational study | Oommen H, Oddbjørn Tveit T, Eskedal LT, Myr R, Swanson DM, Vistad I. | 2021 | Norway | Prospective cohort | 1101 | Intravenous fentanyl administered by midwives starts with bolus doses of 25 µg, administered four times within 20 min (25 µg × 4). After the bolus doses (100 µg), 25 µg can be given every 20 min based on the mother's need for pain relief. The maximum allowable IV fentanyl dose  is 500 µg and the last dose is given no later than 30 min before expected time of birth. | Delivery (mode, induction, postpartum hospital stay), breastfeeding problem, neonatal assessment (apgar, fever) |
| 38 | Bupivacaine Augments Intrathecal Fentanyl for Labor Analgesia | Palmer CM, Maren G Van, Nogami WM, Alves D | 1999 | USA | RCT | 90 | Patients received fentanyl, 25 µg (Elkins-Sinn,Cherry Hill, NJ) | Pain score, maternal assessment (BP, motor, sensory, delivery (duration), analgesia (duration), SE (pruritus), neonatal assessment (FHR, apgar) |
| 39 | The Dose-Response Relation of Intrathecal Fentanyl for Labor Analgesia | Palmer CM, Cork RC, Hays R, Maren G Van, Alves D. | 1998 | USA | RCT | 84 | 5, 10, 15, 20, 25, 35, or 45 µg intrathecal fentanyl was given diluted with preservative normal saline to a total volume of 1.5ml. | Pain score, maternal assessment (BP), analgesia (duration), SE (nausea, vomiting, pruritus, sedation) |
| 40 | Chronobiology of subarachnoid fentanyl for labor analgesia | Pan PH, Lee S, Harris L, Pan PH, Lee S, Harris L. | 2005 | USA | RCT | 70 | Each patient was given a 20µg subarachnoid fentanyl dose, which was prepared with a 1-ml tuberculin syringe, mixed with 0.6-ml normal saline to a  total volume of 1 ml, and then transferred to a 3-ml syringe for subarachnoid injection. | Pain score, maternal assessment (BP, HR, RR, motor, sensory), delivery (mode, contraction), analgesia (duration), SE (vomiting, pruritus), neonatal assessment (FHR) |
| 41 | The addition of hydromorphone to epidural fentanyl does not affect analgesia in early labour | Parker RK, Connelly NR, Lucas T, Faheem U, Rizvi AS, El-Mansouri M, et al. | 2002 | USA | RCT | 43 | the patient was epidural injection of fentanyl 100 µg with normal saline to a total volume of 10 mL | Pain score, maternal assessment (BP, HR, RR, motor, SPO2), delivery (mode), SE (nausea, vomiting, pruritus, sedation), neonatal assessment (FHR) |
| 42 | A comparison of meperidine and fentanyl for labor pain reduction in Phramongkutklao hospital | Raksakulkiat S. | 2019 | Thailand | RCT | 92 | In the fentanyl group, the pregnant women received fentanyl 50 μg intravenously  and additional doses would be given every hour in accordance with maternal request, with a maximum of five doses. | Pain score, maternal assessment (HR, motor), delivery (mode, duration), SE (nausea, vomiting, pruritus, sedation), breastfeeding problem, neonatal assessment (FHR, apgar, birthweight, naloxone requirement) |
| 43 | Randomized Comparison of Meperidine and Fentanyl During Labour | Rayburn WF, Smith CV, Parriott JE, Woods RE. | 1990 | USA | RCT | 105 | 50-100 μg of IV fentanyl every hour | Pain score, SE (nausea, vomiting, pruritus, sedation), neonatal assessment (FHR, apgar, cord blood gases, naloxone requirement, Neurologic & Adaptive Capacity Score) |
| 44 | Fentanyl citrate analgesia during labor | Rayburn W, Rathke A, Leuschen MP, Chleborad J, Weidner W. | 1989 | USA | Quasi-experimental study | 249 | A standard 50 μg or 100 μg dose was administered intravenously as often as every hour on request during active labor (cervical dilation 3 to 10 cm). | Maternal assessment (BP, RR), delivery (mode, duration, induction), analgesia (fentanyl concentration), SE (nausea, sedation), neonatal assessment (FHR, birthweight, naloxone requirement, Neurologic & Adaptive Capacity Score, SPO2) |
| 45 | Epidural fentanyl and perineal pain in labour. Anaesthesia | Reynolds F, O’Sullivan G. | 1989 | United Kingdom | RCT | 46 | Epidural fentanyl 100 µg made up to 10 ml with physiological saline. | Pain score, maternal assessment (BP), delivery (mode, induction), analgesia (duration, additional analgesia), SE (nausea, vomiting, pruritus, sedation, neurological symptoms), satisfaction, neonatal assessment (FHR, apgar) |
| 46 | The effect of the addition of ropivacaine or bupivacaine upon pruritus induced by intrathecal fentanyl in labour | Shah MK, Sia ATH, Chong JL | 2000 | Singapore | RCT | 60 | The patients received intrathecal fentanyl 25 µg and 2ml of 0.9% saline. | Pain score, maternal assessment (BP, HR, RR, motor, sensory), analgesia (duration), SE (nausea, vomiting, pruritus, sedation, shivering), satisfaction, neonatal assessment (FHR) |
| 47 | An intravenous fluid bolus is not necessary before administration of intrathecal fentanyl for labor analgesia | Shannon MT, Ramanathan S. | 1998 | USA | RCT | 30 | Intrathecal fentanyl 25 µg with or without prior administration of IV fluid | Pain score, maternal assessment (BP), neonatal assessment (FHR) |
| 48 | Influence of intravenous fentanyl on fetal biophysical parameters during labor | Smith C V., Rayburn WF, Allen K V., Bane TM, Livezey GT. | 1996 | USA | RCT | 24 | The study group received a standard dose of 50 μg IV  fentanyl (Sublimaze, Janssen Pharm, Piscataway, NJ) | Neonatal assessment (FHR, apgar, cord blood gases, birthweight, congenital anomalies, body movement) |
| 49 | Intrathecal midazolam II: Combination with intrathecal fentanyl for labor pain | Tucker AP, Mezzatesta J, Nadeson R, Goodchild CS. | 2004 | Australia | RCT | 30 | Ten micrograms of intrathecal fentanyl was chosen because of its association with a modest analgesic effect and minimal side effects such as pruritus and nausea. | Pain score, maternal assessment (BP, HR, RR, motor, sensory, induction), analgesia (additional analgesia), SE (nausea, vomiting, pruritus, sedation, headache, neurological symptoms), neonatal assessment (FHR, apgar) |
| 50 | Spinal and epidural sufentanil and fentanyl in early labour | Väänänen A, Kuukasjärvi M, Tekay A, Ahonen J, Vaananen A, Kuukasjarvi M, et al. | 2019 | Finland | RCT | 80 | The spinal groups received 20 μg of fentanyl with normal saline to a total volume of 2mL. The epidural groups received 100 μg of fentanyl diluted to a total volume of 5 mL. | Pain score, delivery (mode, duration), analgesia (duration), SE (nausea, vomiting, pruritus), satisfaction |
| 51 | Epidural fentanyl in labour | Vella LM, Willatts DG, Knott C, Lintin DJ, Justins DM, Reynolds F. | 1985 | United Kingdom | RCT | 40 | Each patient received fentanyl 80 µg, either intravenously over 30 minutes, | Pain score, maternal assessment (BP), delivery (mode, duration), analgesia (fentanyl concentration, additional analgesia), SE (nausea, vomiting, pruritus, neurological symptoms), neonatal assessment (FHR) |
